# *π*–*π* Conjugated Bonds Stacking/Scattering for Switchable Lubrication in Supramolecular Hydrogel

**DOI:** 10.1002/advs.202500447

**Published:** 2025-03-05

**Authors:** Shuhang Deng, Shijia He, Guilong Yan, Li Wang, Zhenyu Li, Jingyu Chen, Jingjuan Lai, Dong Li, Dong Xiang, Chunxia Zhao, Hui Li, Xuezhong Zhang, Han Li, Xungai Wang, Yuanpeng Wu

**Affiliations:** ^1^ The Center of Functional Materials for Working Fluids of Oil and Gas Field School of New Energy and Materials Southwest Petroleum University Chengdu 610500 China; ^2^ Sichuan Engineering Technology Research Center of Basalt Fiber Composites Development and Application State Key Laboratory of Oil and Gas Reservoir Geology and Exploitation Southwest Petroleum University Chengdu 610500 China; ^3^ JC STEM Lab of Sustainable Fibers and Textiles School of Fashion and Textiles Hong Kong Polytechnic University Hong Kong 100872 China

**Keywords:** dual‐responsive, gel‐sol, hagfish, lubrication, mucus

## Abstract

Existing polymer materials often lack the ability to dynamically adjust lubrication in response to external stimuli. This gap in adaptable lubrication technology limits their application in advanced systems such as intelligent motion devices and soft robotics. To address this challenge, a dual‐responsive lubricating hydrogel is introduced designed with a thermal and shear‐responsive supramolecular network integrated into a stable polymer framework. The hydrogel exhibits a partial gel‐sol transition under thermal or shear stress, mimicking the mucus secretion behavior of hagfish. As a result, the hydrogel′s surface secretes a liquid supramolecular layer that significantly alters its coefficient of friction (COF), achieving up to a 20‐fold reduction in friction due to the reversible supramolecular transition. This hydrogel demonstrates significant potential as an intelligent lubrication material capable of dynamically responding to varying stimuli, paving the way for more adaptive and efficient lubrication technologies.

## Graphical abstract

Inspired by the hagfish′s behavior of secreting mucus to escape from predators, a dual‐responsive intelligent lubricating material (PPRA) is successfully prepared. When PPRA is heated or sheared, a partial gel‐sol transition occurs in the hydrogel, resulting in a sol‐state RPAD layer forming on the surface, which acts as a lubricating layer and reduces the coefficient of friction (COF).

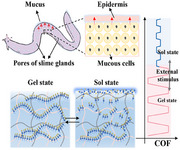

## 1 Introduction

In nature, organisms have evolved organs with friction adaptability for complex environments, inspiring artificial intelligent material development.^[^ ^1^, ^2^, ^3^ ^]^ *Geckos*, for example, achieve dynamic reversible adhesion via their footpads′ unique microstructures, and van der Waals interactions with substrates.^[^ ^4^ ^]^ *Earthworms* secrete mucus in response to external stimuli, with their rough skin of macroscopic gaps and micropores stabilizing the mucus into a thick, surface lubricating layer, reducing resistance and wear.^[^ ^5^, ^6^ ^]^ Inspired by these mechanisms, various intelligent lubrication materials like pH‐responsive materials,^[^ ^7^, ^8^ ^]^ antifouling coatings,^[^ ^9^, ^10^ ^]^ and actuators^[^ ^11^, ^12^ ^]^ have been developed. Biological lubrication, a complex process, is influenced by surface properties and internal mucus secretion.^[^ ^13^, ^14^ ^]^ However, current research primarily focuses on modifying surface chemical composition,^[^ ^15^, ^16^ ^]^ topography,^[^ ^17^, ^18^ ^]^ and mechanical properties,^[^ ^19^ ^]^ with the creation of materials that secrete lubricants still posing a significant challenge.

**Figure 1:**
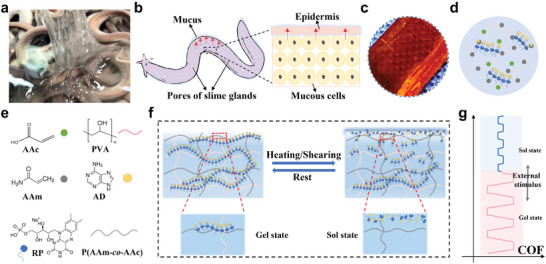
a) Photograph of the hagfish, b) schematic diagram illustrating the secretion of mucus of the hagfish, c) image of the hydrogel slice, d) e) schematic of the hydrogel fabrication procedure, f) g) the possible lubricating mechanism of the prepared thermal/shear‐responsive lubricating hydrogel.

Hagfish immediately secrete mucus in response to predator stimulation, including mechanical and light stimuli (**Figure** **1**a).^[^ ^20^, ^21^ ^]^ Specifically, the mucus cells beneath the epidermis secrete mucus, which is then released through the mucus gland pores (Figure 1b). The secreted mucus, a type of highly hydrated biomacromolecule on their skin,^[^ ^22^ ^]^ serves multiple functions: reducing friction to evade predators, decreasing swimming drag, and facilitating adaptation to aquatic environments. Supramolecular hydrogels, which can transition from gel to sol under various external stimuli like light,^[^ ^23^ ^]^ shear forces,^[^ ^24^ ^]^ temperature,^[^ ^25^ ^]^ and pH,^[^ ^26^ ^]^ have shown potential in this area. A recent innovative approach involves integrating non‐covalent supramolecules with covalent networks, deemed effective for creating hydrogels that replicate natural secretion capabilities.^[^ ^27^, ^28^ ^]^

Herein, we reported a dual‐responsive intelligent lubricating material (PPRA), comprising a stimuli‐responsive supramolecular component and a stable polymer network. The P(AAm‐*co*‐AAc)/PVA, due to its excellent mechanical properties, serves as a stable framework.^[^ ^29^ ^]^ RPAD,^[^ ^30^ ^]^ a typical thixotropic and thermal‐responsive supramolecular hydrogel, undergoes a rapid gel‐sol transition upon heating or shearing due to the disruption of weak noncovalent interactions such as hydrogen bonds and *π*–*π* stacking. When the PPRA was heated or sheared, a partial gel‐sol transition occurred in the hydrogel, resulting in a sol‐state RPAD layer forming on the surface, which acted as a lubricating layer and reduced the coefficient of friction (COF).

## 2 Results and Discussion

### 2.1 Concept and Construction of the PPRA Hydrogel

In this work, the thermal and shear responsive hydrogels were prepared by a simple method, as shown in Figure 1d, e, resulting in the formation of three interpenetrating networks within the hydrogel: the P(AAm‐*co*‐AAc) and PVA covalent networks, along with the supramolecular RPAD non‐covalent network. The image of the hydrogel slice is shown in Figure 1c. The robust P(AAm‐*co*‐AAc)/PVA covalently crosslinked double network acts as the framework for the entire hydrogel. The flexible supramolecular RPAD network intertwines with the interpenetrating P(AAm‐*co*‐AAc) and PVA covalent networks. Based on the concentration of RPAD in P(AAm‐*co*‐AAc)/PVA hydrogels, the hydrogels were marked as PPRA‐1, PPRA‐2, PPRA‐3, PPRA‐4, PPRA‐5.

The prepared PPRAs were entangled interpenetrating networks, which were the thermal and shear responsive supramolecular RPAD non‐covalent network, and P(AAm‐*co*‐AAc) and PVA double interpenetrating networks. The fibrillar supramolecular network of RP and AD were physically cross‐linked through via H─bonding and supramolecular organization through *π*–*π* stacking process.^[^ ^34^, ^35^, ^36^ ^]^ The RPAD supramolecular hydrogel showed thermo‐reversible and thixotropic behaviors (Figure S1, Supporting Information). The RPAD melting temperature is ≈50 °C, which was measured by the dropping ball method. When the hydrogel was heated or subjected to shear force, the RPAD supramolecular network disassembled due to weak non‐covalent. The gel state of RPAD transferred to the sol state containing RP, AD monomers, and RPAD oligomers.^[^ ^38^ ^]^ Then, the sol state supramolecular release to the hydrogel surface and forms a lubricating layer to reduce friction (Figure 1f,g). When the PPRA hydrogel was rested, RP again assembled with AD to gel.

### 2.2 Characterizations and Mechanical Properties of the PPRA Hydrogel

**Figure 2:**
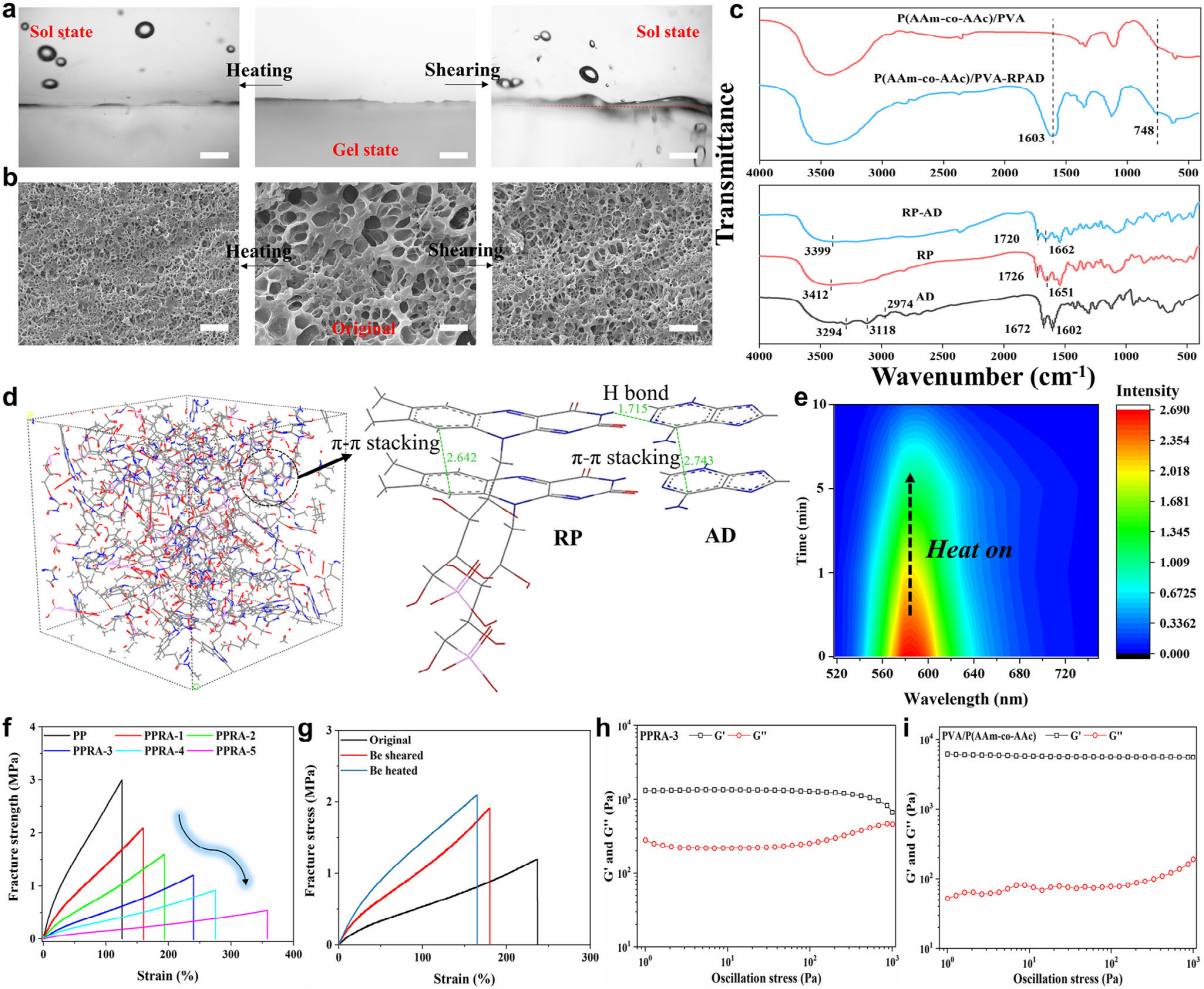
a) Profiled images of PPRA‐3 hydrogel, original, after being sheared and after being heated, b) SEM images of PPRA‐3 hydrogel, original, after being sheared and after being heating, c) The FTIR spectra of P(AAm‐*co*‐AAc)/PVA, P(AAm‐*co*‐AAc)/PVA‐RPAD, RP, AD, and RPAD, d) Molecular dynamic and DFT results of hydrogel, e) fluorescence intensity of the sol layer extract on PPRA‐3 as a function of the heating time 0 to 10 min, f) tensile stress‐strain curves of P(AAm‐*co*‐AAc)/PVA and PPRA hydrogels, corresponding, g) tensile stress‐strain curves of PPRA‐3 hydrogel, original, after being sheared and after being heating, h) the oscillation stress dependency of the G′ and G″ of PPRA‐3, (i) the oscillation stress dependency of the G′ and G″ of P(AAm‐*co*‐AAc)/PVA.

To identify the lubricating layer on PPRA induced by the gel‐sol transition, direct profiling observations of the hydrogel surface post‐heating and post‐shearing were conducted. Hydrogel slices were prepared using paraffin sections. No sol layer was observed on the original hydrogel surface, but after heating or shearing, a transparent sol layer became visible (**Figure** **2**a). This is evident in the photographs (Figures S2–S4, Supporting Information), where the sol layer is noticeable on the hydrogel surface. The disassembly of the RPAD supramolecular network results in this sol layer formation, making surface wettability a key aspect in understanding the hydrogel′s lubricating mechanism.^[^ ^37^, ^38^ ^]^ The wettability of both PPRA (Figure S5, Supporting Information) and PVA/P(AAm‐*co*‐AAc) hydrogels (Figure S6, Supporting Information) was measured. The contact angle (CA) on PPRA was 60.2 ± 1.8°, decreasing to 7.6 ± 0.7° and 12.4 ± 1.1° post‐heating and post‐shearing, respectively, due to the sol layer formation. The CA did not reach 0°, possibly because the sol layer was too thin to induce a super hydrophilic state. The PVA/P(AAm‐*co*‐AAc) hydrogel showed no significant wetting changes. These results indicate that the RPAD plays a key role in the responsive lubrication properties of the hydrogel. Figure 2b presents the scanning electron microscopy (SEM) images of the hydrogel in different states. Typical loose and porous structures were observed on the original PPRA‐3 hydrogel. Intriguingly, upon heating and shearing, the surface structures became more porous, resembling the PVA/P(AAm‐*co*‐AAc) hydrogel′s structure post freeze‐drying (Figure S7, Supporting Information). This suggests the disassembly of RPAD within the hydrogel.

The Flourier Transformed Infrared Spectroscopy (FTIR) was used to confirm the components of PPRA (Figure 2c). The peak that appeared at 1602 cm^−1^ was the stretching vibration signal of the carbon‐nitrogen double bond (─C═N),^[^ ^30^ ^]^ the peak at 748 cm^−1^ was attributed to the benzene ring,^[^ ^39^ ^]^ indicating the successful fabricating of PPRA hydrogel. The absorption band at 3412 cm^−1^ in RP is associated with the ─OH group of the ribityl chain, which shifts to 3399 cm^─1^, indicating that hydrogen bonding occurs during gelation. Similarly, the C═O stretching vibration in RP, originally at 1726 cm^−1^, decreases to 1720 cm^−1^, suggesting hydrogen bonding between the C═O group and the amino/imino functionalities of AD. The ‐NH_2_ and ‐NH stretching frequencies of AD are observed at 3294 cm^−1^ (symmetric), 3118 cm^−1^ (asymmetric), 1672 cm^−1^ (bending), and 2974 cm^−1^ (stretching). In the RPAD, the intensities and positions of these peaks are diminished or shifted to lower frequencies, indicating the participation of these functional groups in hydrogen bonding between RP and AD. Additionally, the C═N and C═C stretching modes of AD, along with the aromatic C═C stretching mode of RP, are detected at 1602 and 1651 cm^−1^, respectively. In contrast, the RPAD shows a single peak at 1662 cm^−^¹, likely reflecting a significant non‐covalent (*π*–*π*) interaction between the two molecules during the gelation process.

**Figure 3:**
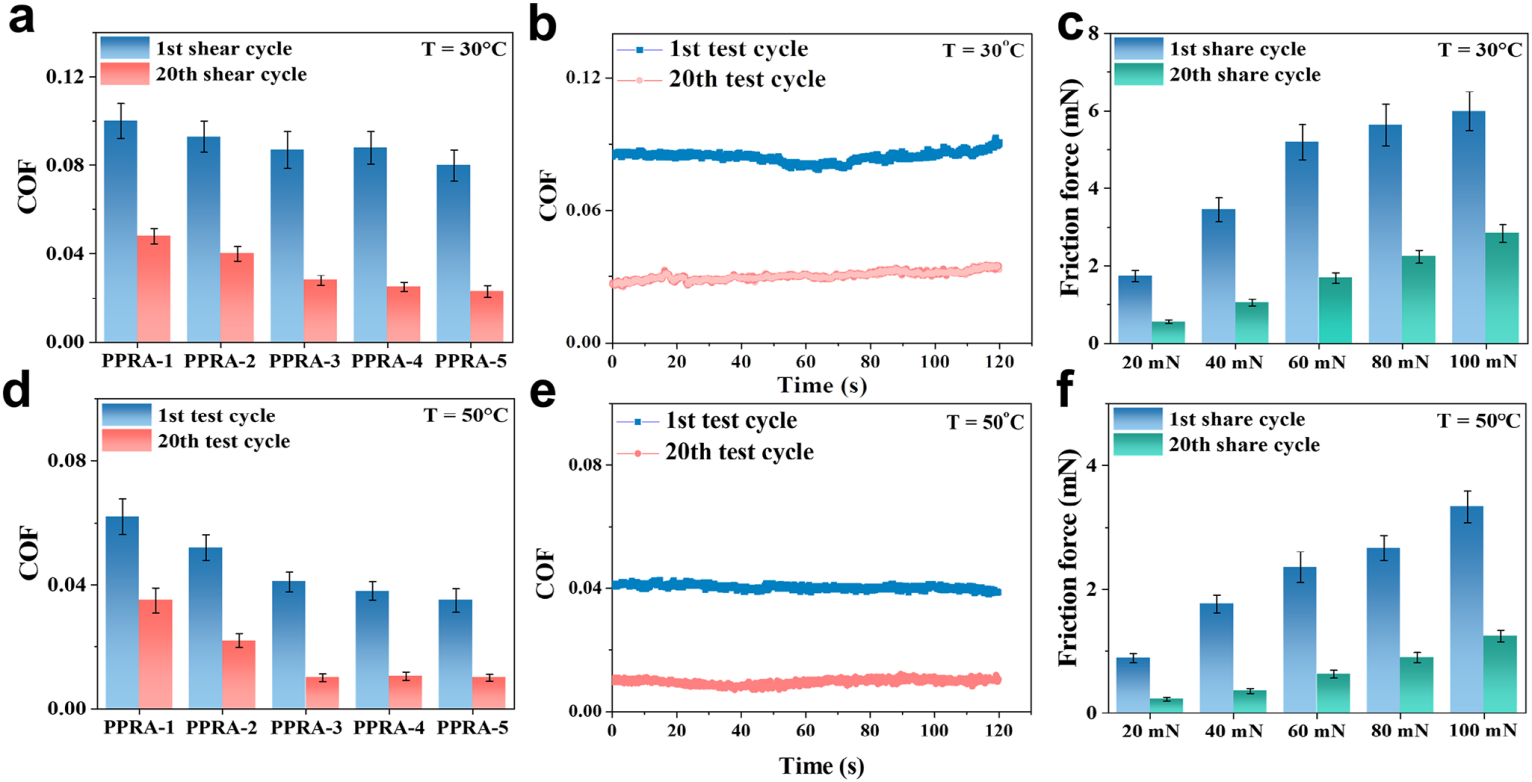
a, d) COF on the PPRA hydrogels with different RPAD concentrations (Load: 60 mN, shear velocity: 0.1 mm s^−1^), b, e) corresponding COF curves of (a) and (d), c, f) keeping the sliding velocity at 0.1 mms ^−1^, the relationship between the friction force on PPRA‐3 under different loads of 20–100 mN.

The Cohesive Energy Density (CED) calculated through molecular dynamics simulation is 776.1 J cm^−3^, indicating strong interaction forces within the system (Figure 2d). To further elucidate the thixotropic and thermal‐responsive behavior of the RPAD supramolecular hydrogel, density functional theory (DFT) was employed to calculate the energy of model molecules. The DFT calculations revealed significant energy differences between RP, AD, and RPAD (Table S3, Supporting Information). At 293 K (**Table** **1**), the interaction energy of RPAD was −54.45 kcal mol^−1^, while they were −4.32 kcal mol^−1^ (T = 293 K, be sheared) and ‐3.06 kcal mol^−1^ (T = 323 K). This indicates that the interaction energy within the RPAD supramolecular network decreases, due to the breaking of hydrogen bonds and the disassembly of *π*–*π* stacking between RP and AD. Thus, the DFT calculations confirm that the RPAD supramolecular network disassembles when heating or shearing, aligning with the experimental results. RPAD supramolecules are also known to exhibit fluorescent properties due to the isoalloxazine rings of riboflavin in their chains, with an excitation wavelength of 589 nm. The PPRA hydrogel was assessed using a fluorescence spectrum (excitation at 373 nm) in solid mode, post‐heating, and shearing. An observed decrease in fluorescence intensity with prolonged heating and shearing (Figure 2e; Figure S8, Supporting Information) suggests increased RPAD disassembly over time, from 0 to 10 min. This aligns with the coefficient of friction (COF) depicted in **Figure** **3**b.

**Table 1:** The interaction energy of RPAD.

Model	Interaction energy [kcal/mol, T = 293 K]	Interaction energy [kcal/mol, T = 293 K, be sheared]	Interaction energy [kcal/mol, T = 323 K]
RPAD	−54.45	−4.32	−3.06

The tensile test was carried out to assess the effect of RPAD on the mechanical properties of PPRA hydrogels, are shown in Figure 2f. The fracture strength of PVA/P(AAm‐*co*‐AAc) hydrogel was ≈3 MPa, while the fracture strength of PPRAs decreased with the increase in RPAD supramolecular contents. The stresses of hydrogels decreased from 3 ± 0.24 to 0.54 ± 0.05 MPa, with increasing RPAD contents (Figure S9, Supporting Information). The toughness of PPRA decreased from 2136 ± 80 to 936 ± 40 kJ m^−3^ (Figure S10, Supporting Information). This result may be explained by the fact that the easily destroyed non‐covalent supramolecular network and a relatively looser network than the PVA/P(AAm‐*co*‐AAc) hydrogel network.^[^ ^40^ ^]^ Additionally, the seepage of disassembled RPAD implies a reduction in its quantity within the hydrogel. Consequently, based on the mechanical results (Figure 2f), an enhancement in mechanical strength can be inferred. To verify this analysis, the mechanical property of PPRA‐3 hydrogel was tested with different heating/shearing times (Figure S11, Supporting Information). As shown in Figure 2g and Figure S12 (Supporting Information), the fracture strength of PPRA‐3 at 50 °C (2.1 ± 0.14 MPa) was higher than that of at 25 °C (1.23 ± 0.07 MPa). Moreover, the fracture strength (1.92 ± 0.13 MPa) of PPRA‐3 hydrogel (being sheared) became higher than that (1.23 ± 0.07 MPa) of the unsheared PPRA‐3 hydrogel. These results also indicate the RPAD supramolecular would disassemble and the RPAD wept out on the hydrogel surface. The following rheological measurement was conducted to gain insights into the hydrogel′s rheological properties. The storage modulus (G′) indicates the quantity of stored energy in the system, it will characterize the solid‐liked property of the hydrogel, while the loss modulus (G″) represents the dissipated energy, indicative of its liquid‐like properties. Figure S13 (Supporting Information) shows the dynamic frequency sweep experiment of the supramolecular gel at a constant strain of 1%, where the broad linear viscoelastic region with frequency and significantly higher G′ than G″ confirms the gel nature of the RPAD system. For the RPAD supramolecular hydrogel, a thixotropic gel‐sol transition occurs when shear stress reaches 37 Pa (Figure S14, Supporting Information), where G′ and G″ converge. Given that the shear stress at the gel‐sol transition is 37 Pa, the calculated shear force at this point is 3.49 mN, as determined by Formula S2 (Supporting Information). This indicates that FT will disassemble if the shear force exceeds 3.49 mN. When RPAD was introduced into the polymer network, PPRA‐3 hydrogel was sensitive to shear (Figure 2h). In contrast, for the PVA/P(AAm‐*co*‐AAc) hydrogel, the consistently higher G′ compared to G″ indicates an absence of thixotropy (Figure 2i). As demonstrated in Figures S15 (Supporting Information), and 16, G′ decreased after heating and shearing, a trend observed across all prepared hydrogels. In addition, all the other PPRAs were conducted rheological experiments (Figure S17, Supporting Information).

### 2.3 Shear and Thermal Responsive Lubrication on the PPRA Hydrogels

In a demonstration experiment, two pieces of hydrogel were placed on the inclined plane. Without shear force, a weight placed on them did not slide (Figure S18a, Supporting Information). However, when the PPRA hydrogel was sheared, the weight slid until it reached the unsheared hydrogel (Figure S18b and Video S1, Supporting Information). After allowing the PPRA‐3 hydrogel to stand for 10 min, the weight remained immobile, demonstrating the impact of shearing on the hydrogel's properties (Figure S18c, Supporting Information). As shown in Figure S19 (Supporting Information), a reciprocating shear motion is applied to the PPRA surface to test its shear‐responsive lubrication performance. The shear‐responsive lubricating process demonstrated excellent reversibility, maintaining consistent COFs across multiple cycles (Figure S20, Supporting Information). Moreover, the COF was investigated as a function of the RPAD concentration in the hydrogel. The dual‐responsive lubrication phenomenon was consistent across all PPRAs with varying RPAD concentrations (Figure 3a,d), indicating that the RPAD played an important role in the dual‐responsive lubrication. Compared to PPRA hydrogel, the COF of PVA/P(AAm‐*co*‐AAc) hydrogel was stable following the test cycles (Figures S21 and S22, Supporting Information), highlighting the significant role of RPAD supramolecular contents in responsive lubrication. In addition, Figure 3b,d show the corresponding friction curve of the PPRA‐3 hydrogel. To further investigate the responsive lubrication behaviors of the PPRA hydrogels, subsequent experiments will adjust the loading force and shear velocity. Maintaining the shear velocity at 0.1 mms ^−1^, the PPRA's friction force increased with the load from 20 to 100 mN (Figure 3c,f). This increase is attributed to the enlarged hydrogel deformation from the heightened loading force, which expands the contact area between the hydrogel surface and the contact ball.^[^ ^41^ ^]^ This phenomenon aligns with the classical friction formula *f = µF* (where *f* is the friction force, *μ* is the friction coefficient, and *F* is the load), indicating that surface friction escalates with increased load. Moreover, according to the repulsion‐adsorption model,^[^ ^42^ ^]^ the gel's friction force can be expressed as *f = ηvS/h* (where *η* is the sol viscosity between the hydrogel and the contact pair, *v* is the contact pair velocity, *S* is the contact area, and *h* is the sol layer thickness). Assuming the presence of a thin water layer on the hydrogel surface, and considering *h* as constant, the contact area (*S*) between the hydrogel surface and the contact pair increases with the load due to the hydrogel's enhanced deformation from the load increase. When the load was fixed at 60 mN and the shear velocity of the contact pair varied from 0.1 to 0.5 mms ^−1^, the friction force was found to be proportional to the shear velocity (Figure S23, Supporting Information). In this scenario, *η*, *S*, and *h* were considered constant, and the friction force increased with the shear velocity.

**Figure 4:**
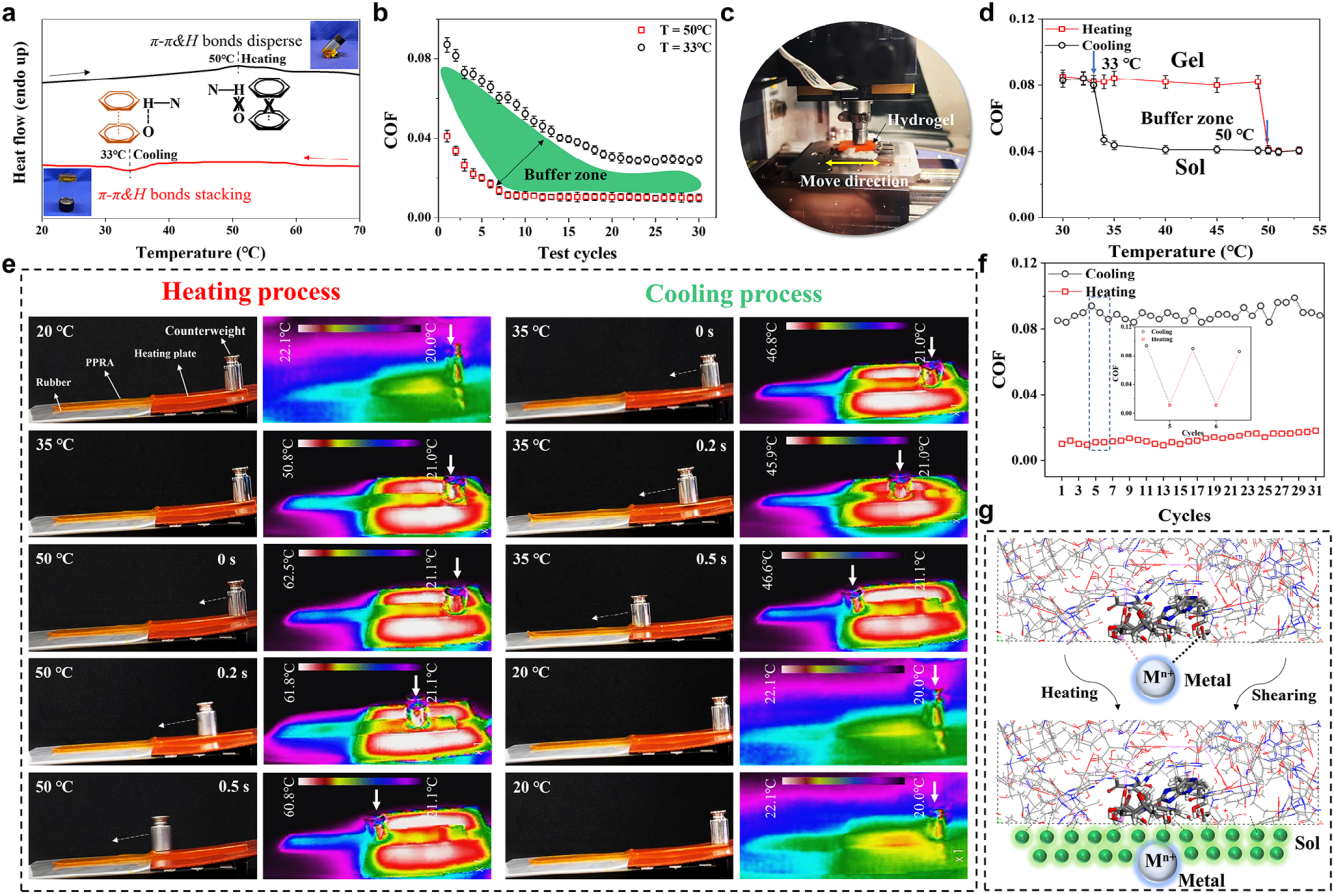
a) DSC thermograms of the RPAD, b) the COFs on the PPRA‐3 hydrogel following 30 test cycles, under different temperatures, c) photograph of friction test, d) thermal‐responsive lubricating behavior of PPRA‐3, e) demonstration of thermal‐responsive lubricating behavior of PPRA‐3, f) reversibility of thermal‐responsive lubricating on PPRA‐3, g) lubricating mechanism of the PPRA hydrogel.

As shown in **Figure** **4**a, RPAD exhibited an endothermic peak at 50 °C and an exothermic peak during cooling, with a hysteresis of ≈17 °C, indicating a first‐order phase transition. Consequently, PPRA's responsive lubrication was examined using a typical sphere‐to‐disk contact mode in a reciprocating sliding test (Figure 4c). A steel ball with a 10 mm diameter reciprocated linearly over the hydrogel surface, under a controlled sliding velocity and load force. In Figure 4b, as the number of test cycles increased, the COF decreased from 0.087 ± 0.003 to 0.029 ± 0.002 (33 °C). This is because friction testing is inherently a repetitive shear process, when the contact ball slides on the surface of the hydrogel, the thixotropic RPAD supramolecular network would disassemble. The RPAD in the gel state would transform into the sol state exuded to the surface, and then form a lubricating layer between the surface of the hydrogel and the contact ball. At 50 °C, the COF decreased more rapidly and eventually stabilized at 0.009 ± 0.001. This behavior is attributed to the limited shear force range, which only affects the surface and subsurface of the PPRA, causing the RPAD to decompose. When the temperature reached 50 °C, RPAD inside the PPRA also decomposed, and under the combined effect of shear load, more sol‐like RPAD was exuded to the surface, resulting in a lower COF. As shown in Figure 4d, as the temperature gradually increased to 50 °C, the COF sharply decreased to 0.041 ± 0.002. When the temperature was gradually lowered, the COF increased sharply to 0.08 ± 0.004 at 33 °C. The unique responsive lubrication ability of PPRA enabled its potential application as one kind of intelligent device. As a demonstration, one hydrogel was placed on a heating plate, while the other was placed on a rubber plate (Figure 4e). Initially, a weight remained stationary due to high static friction and the absence of a lubrication layer between it and the hydrogel. When the PPRA hydrogel was heated to 50 °C, the weight began to slide under the influence of gravity, stopping upon reaching the unheated hydrogel (Video S2, Supporting Information). Interestingly, when the temperature of the PPRA hydrogel dropped to 35 °C, the weight could still slide off from a height. However, as the temperature decreased further, the weight remained stationary, illustrating the reversibility of the RPAD supramolecular gel′s gel‐sol transition. At the same time, the thermal‐responsive lubrication demonstration aligns with the results shown in Figure 4d. Additionally, the thermal‐responsive lubricating process demonstrated excellent reversibility, maintaining consistent COFs across multiple cycles (Figure 4f). Moreover, as shown in Figure 4g, when PPRA was heated or sheared, a partial gel‐sol transition occurred in the hydrogel, resulting in a sol‐state RPAD layer forming on the surface, which acted as a lubricating layer.

To demonstrate the universality of constructing dual‐responsive hydrogel, RPAD supramolecular network was constructed as responsive sections in a series of polymer frameworks, including poly(acrylamide) (PAAm), poly(vinyl alcohol) (PVA), poly(hydroxyethyl methacrylate) (PHEMA), poly(acrylic acid) (PAAc), poly(meth acrylamide) (PMAM) (the details in supporting information). These hydrogels also exhibited thermal/shear‐responsive lubricating properties (Figure S24, Supporting Information). These results indicate that introducing a thermal/shear‐responsive supramolecular network into the polymer network is a universal strategy for constructing a thermal/shear‐responsive hydrogel.

### 2.4 Demonstration and Application of PPRA Hydrogel

**Figure 5:**
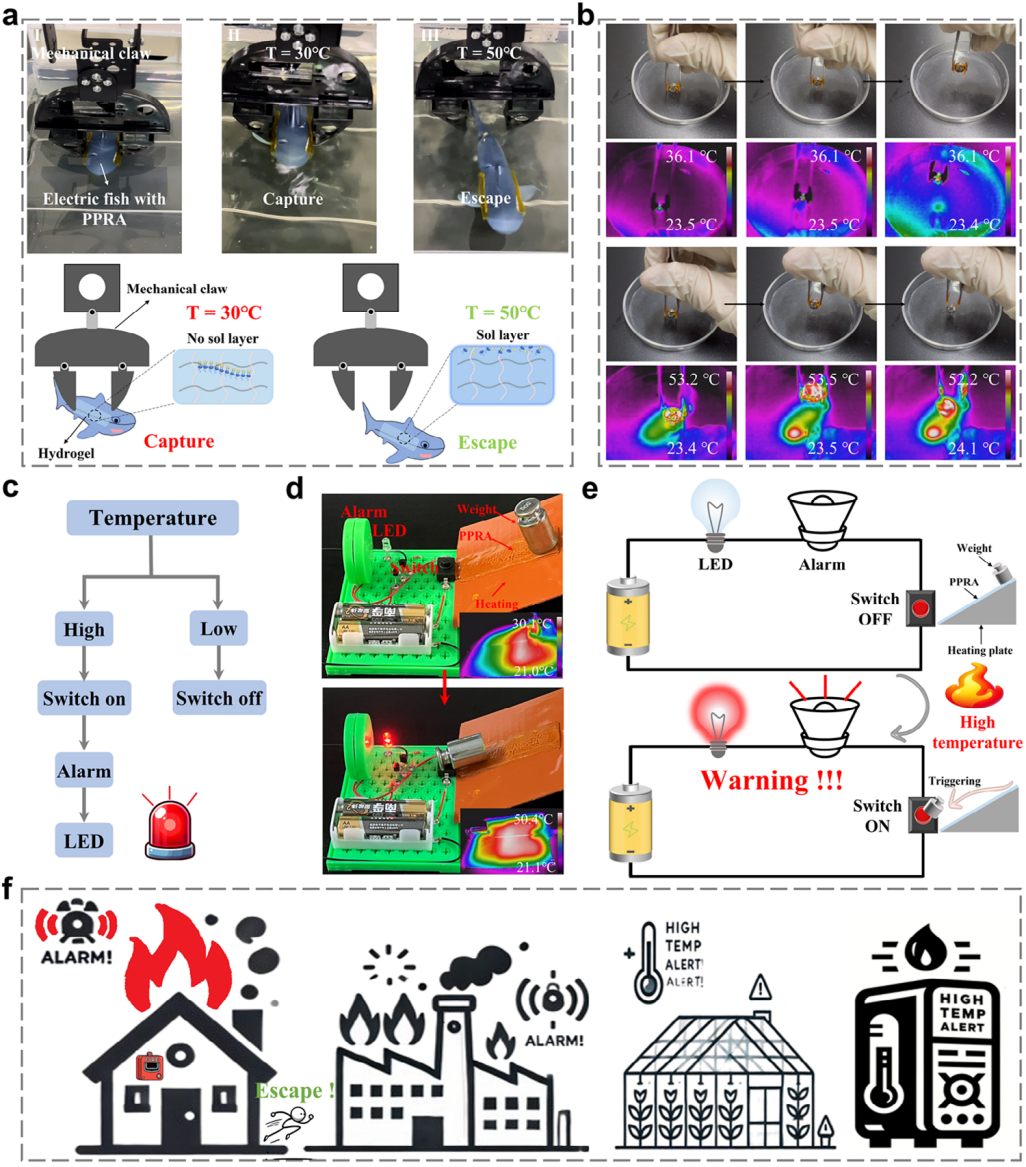
a) Demonstration of the underwater mechanical system to in situ capture device, b) demonstration of temperature identification, c) Diagram of the monitoring process, d) demonstration of the fire warning device based on PPRA hydrogels, e) schematic diagram of the fire warning device, f) device was used for daily home fire alarms and broad potential applications.

To demonstrate the ability of the PPRA hydrogel to regulate friction similarly to a hagfish, an in situ capture device was constructed using a mechanical claw and an electric fish. The PPRA was adhered to both sides of the electric fish with glue (Figure S25, Supporting Information), and the electric fish was then grasped by a mechanical claw under a constant load (≈300 mN). As shown in **Figure** **5**a, in 30 °C water bath, the electric fish was effectively captured by the mechanical claw and could not swim forward (Video S3, Supporting Information). However, in 50 °C water bath, the electric fish managed to escape (Video S4, Supporting Information). This escape was facilitated by the lubricant secreted by the PPRA at high temperatures. These demonstrations mimic the escape behavior of a hagfish, showing that PPRA can function as an intelligent switchable material to control device motion. Furthermore, due to its temperature‐responsive lubrication properties, the PPRA hydrogel can be used for temperature identification. As a demonstration, two pieces of PPRA were attached to the tips of tweezers. When the steel ball was at 30 °C (Figure 5b), it could be easily picked up by the tweezers due to high friction, and it did not fall off even when the tweezers were shaken. However, when the steel ball was heated to 50 °C (Figure 5b), it quickly dropped after being picked up, due to the decomposition of RPAD.

The unique thermal‐responsive lubricating properties of hydrogels enabled its potential application as a fire‐alarm system, the monitoring process is shown in Figure 5c. The heat‐responsive lubricating properties of the PPRA hydrogel enable a weight to slide and trigger a switch, which controls a circuit connected to an alarm and an LED, thus serving as a fire warning mechanism. A heating plate was used as a heat source to simulate high‐temperature conditions, such as those in fire incidents. As the heating plate temperature rises, the hydrogel surface secretes a sol layer, reducing friction and allowing the weight to slide off the heating plate, thereby triggering the switch, lighting up a bulb, and activating a buzzer alarm (Figure 5d,e; Videos S5 and S6, Supporting Information). Hence, this device can be used for daily home fire alarms. In addition, this thermal‐responsive switch design has broad potential applications in factory fire alarms, greenhouse temperature monitoring, and electronic device overheating protection (Figure 5f).

## 3 Conclusion

In summary, inspired by the hagfish′s behavior of secreting mucus to escape from predators, a dual‐responsive intelligent lubricating material (PPRA) was successfully prepared. Due to the thermal/shear‐responsive capability of RPAD supramolecular hydrogel, PPRA could realize a switchable surface state from a high friction state (μ≈0.16) to super‐lubricating state (μ≈0.008). The introduction of a thermal/shear‐responsive supramolecular into a polymer network is a universal strategy for constructing hagfish‐inspired responsive hydrogels. As an intuitive demonstration of its application potential, PPRA can be used for smart motion devices and fire alarm systems. The PPRA may show more promising applications in novel smart devices far beyond intelligent lubrication systems, such as soft robotics, and anti‐fouling.

## 4 Experimental Section

### Materials and Chemicals

Poly(vinyl alcohol) (PVA) (degree of hydrolysis 99%, M_w_ = 88 000), acrylamide (AAm) (99%), and acrylic acid (AAc) (99%) were manufactured by Sigma–Aldrich Co., Ltd. Riboavin‐5′‐phosphate sodium salt (RP) (99%), adenine (AD) (98%), and glutaraldehyde (GD) (25%) were purchased from Shanghai Aladdin Bio‐chem Technology Co., Ltd. Phosphate buffer saline (PBS) (pH = 4) was purchased from KeLong Chemical Co., Ltd. N,N′‐methylenedi‐acrylamide (MBAA) and potassium persulfate (KPS) were purchased from Sinopharm Chemical Reagent Co., Ltd. Distilled water was made in the laboratory.

### Preparation of Hydrogels

The hydrogels were synthesized by a facile one‐pot one‐step reaction. In brief, 1.42 g AAm and 0.72 g AAc were completely dissolved in 5 mL PBS solution and then added to 5 mL pre‐dissolved 50 mg mL^−1^ PVA solution. Subsequently, the designated quantities of RP and AD (1:1 mol ratio) were dissolved in the mixture solution at 80 °C. Then 0.003 m of MBAA, KPS, and 44.3 µL GD were added to the mixture solution. After 6 h gelation, the prepared P(AAm‐*co*‐AAc)/PVA‐RPAD hydrogels were stored for further experiments. Based on the concentration of RPAD in hydrogels (1.0, 2.0, 3.0, 4.0, and 5.0 mg mL^−1^) in the experiment, the prepared hydrogels were marked as PPRA‐1, PPRA‐2, PPRA‐3, PPRA‐4 and PPRA‐5.

### Mechanical Measurements

A 200 N load cell equipped Instron machine (Instron 5965, Norwood, MA) was used to examine the mechanical properties of hydrogels. The hydrogel blocks were sliced into a dumbbell shape (30 mm × 3 mm × 2 mm) and stretched at 50 mm min^−1^ for tensile testing.^[^ ^31^ ^]^

### Rheological Measurements

The rheological behaviors of the hydrogels were analyzed with a strain/stress‐controlled rheometer (AR 2000, TA Instrument, USA). The diameter of the steel plate was 30 mm with a gap of 1 mm and a cone angle of 4 degrees. The frequency (f) sweep test was carried out at 25  C. With a diameter of 35 mm and a thickness of 2 mm, samples are fabricated in the shape of cylindrical objects. The G′ (storage modulus) and G′′ (loss modulus) of the hydrogels were calculated by taking the values at a particular angular frequency of 10 rad s^−1^ for the oscillation mode at room temperature and the shear stress increased from 1 Pa to 10 000 Pa.

### Friction Test

The coefficient of friction under different conditions of hydrogels was measured on a multi‐functional friction and wear ball‐on‐disk reciprocating tribometer (UMT‐2, Bruker, USA). A steel ball with a diameter of 10 mm was used as a friction pair. In the friction experiment the temperature was selected as 30 °C to investigate the shear‐responsive lubricating property, moreover, the temperature was selected as 50 °C to investigate the thermal‐responsive lubricating property. The shearing velocities were 0.1, 0.2, 0.3, 0.4, and 0.5 mms ^−1^, and the load force were 20, 40, 60, 80, and 100 mN. One reciprocating rectilinear motion was marked as one test cycle, the distance of one cycle was 6 mm. The results were the average value of three repetitions of the experiment. In addition, the contact pressure between and contact ball can be calculated by the Hertzian contact mechanism (Formula S1, Supporting Information). Corresponding to a range of loads *F_n_* and various mean contact pressures *P* (Table S1, Supporting Information), to obtain the COFs of hydrogels.

### The Morphology Characterization

SEM was performed to research the surface morphologies of the hydrogel. A Zeiss EVOMA 15 microscope equipped with an energy dispersive x‐ray spectrum (EDS) was used to obtain scanning electron microscopy (SEM) images.^[^ ^32^, ^33^ ^]^

### Fluorescence Spectrum

The fluorescence study of the hydrogels was carried out on RF‐5301 PC Spectrophotometers (Shimadzu, Japan) in a solid mode. Each gel sample in a quartz cell of 1 cm path length was excited at 373 nm and the emission scans were recorded from 390 to 700 nm using a slit width of 5 nm with a 1 nm wavelength increment having an integration time of 0.1 s.

### Density Functional Theory (DFT) Calculations

To gain further insight into the molecular interactions of the materials, density functional theory (DFT) calculations were carried out. The simulation was performed using the DFT program DMol^3^ in Material Studio 8.0 (Accelrys, San Diego, CA). The interaction energy (E_i_) could be used to describe the interaction intensity of the components in the system. Specifically, E_i_ is calculated by the difference between the total energy of the composite system (E_t_) minus the summation of the energy of each component in the system (ΣE_c_), which could be defined as E_t_‐ΣE_c_. For each of the investigated compounds the energy of its monomer and dimer was calculated after optimization of their molecular geometry.

### Molecular Dynamic Analysis

For Molecular Dynamic (MD) Calculations, the PVA, P(AAm‐*co*‐AAc), RP, and AD were put into a periodic cell by the Amorphous Cell Construction of Materials Studio 8.0. The number of configurations was 100, and the density of the final configurations was 1 g·cm^−3^. The forcefield type was the COMPASS and the system temperature was set as 298.0 K.

## Conflict of Interest

The authors declare no conflict of interest.

## Supporting information



Supporting Information

Supplemental Video 1

Supplemental Video 2

Supplemental Video 3

Supplemental Video 4

Supplemental Video 5

Supplemental Video 6

## Data Availability

The data that support the findings of this study are available from the corresponding author upon reasonable request.
