# Supplementary material for: π–π Conjugated Bonds Stacking/Scattering for Switchable Lubrication in Supramolecular Hydrogel
Source: Adv Sci (Weinh). 2025 Mar 5;12(16):2500447. doi: 10.1002/advs.202500447 (PMC12021116; doi:10.1002/advs.202500447)
Supplement: Supplementary file 1 — Supporting Information [file ADVS-12-2500447-s004.docx]

Supporting Information

**π-π conjugated bonds stacking/scattering for switchable lubrication in supramolecular hydrogel**

*Shuhang Deng, Shijia He, Guilong Yan* , Li Wang*, Zhenyu Li, Jingyu Chen, Jingjuan Lai, Dong Li, Dong Xiang, Chunxia Zhao, Hui Li, Xuezhong Zhang, Han Li, Xungai Wang, Yuanpeng Wu**

S. Deng, S. He, G. Yan , L. Wang, Z. Li, J. Chen, J. Lai, D. Li, D. Xiang, C. Zhao, H. Li, X. Zhang, Y. Wu

The Center of Functional Materials for Working Fluids of Oil and Gas Field,

School of New Energy and Materials, Southwest Petroleum University,

Chengdu, 610500(China)

E-mail: ypwu@swpu.edu.cn, [guilong.yan@swpu.edu.cn](mailto:guilong.yan@swpu.edu.cn), [wangli@swpu.edu.cn](mailto:wangli@swpu.edu.cn)

G. Yan , L. Wang, Z. Li, J. Chen, J. Lai, D. Li, D. Xiang, C. Zhao, H. Li, X. Zhang, Y. Wu

Sichuan Engineering Technology Research Center of Basalt Fiber Composites Development and Application,

State Key Laboratory of Oil and Gas Reservoir Geology and Exploitation,

Southwest Petroleum University,

Chengdu, 610500(China)

E-mail: ypwu@swpu.edu.cn, [guilong.yan@swpu.edu.cn](mailto:guilong.yan@swpu.edu.cn), [wangli@swpu.edu.cn](mailto:wangli@swpu.edu.cn)

H. Li, X. Wang

JC STEM Lab of Sustainable Fibers and Textiles, School of Fashion and Textiles,

Hong Kong Polytechnic University,

Hong Kong, 100872(China)

**Materials and methods:**

1. Materials
2. RPAD supramolecular incorporation in different hydrogels
3. Measurement of gel-sol transition temperature
4. DSC analysis
5. Fourier transform infrared spectroscopy (FTIR)
6. Static water contact angle (θ)
7. **Materials**

Acrylamide (AAm), acrylic acid (AAc), 2-hydroxyethyl methacrylate (HEMA), poly(vinyl alcohol) (PVA) and methacrylamide (MAM) were purchased from Aladdin (China). N, N, N′, N′-tetramethyl ethylenediamine (TMEDA), potassium persulfate (KPS), 2-Hydroxy-4'-(2-hydroxyethoxy)-2-methylpropiophenoneand (Irgacure 2959) N, N’-methylene bisacrylamide (MBAA) were purchased from Sigma-Aldrich (China).

1. **Hydrogel preparation**

P(AAm-co-AAc)/PVA-RPAD hydrogel described in the main text, the effect of supramolecular RPAD-incorporation on friction was examined in different hydrogels. RPAD supramolecular was incorporated, at concentrations in the range 1-5 mg/mL, in several other hydrogels.

A) Polymethacrylamide hydrogel (PMAM; 0.003 M MBAA, 0.003 M KPS, 2 M MAM, 10 mL PBS solution; 40℃ 4h).

B) Poly(acrylamide) hydrogel (PAAm; 0.003 M MBAA, 0.003 M KPS, 3 M AAm, 10 mL PBS solution; 40℃ 4h).

C) Poly(hydroxyethyl methacrylate) hydrogel (PHEMA; 0.003 M MBAA, 0.004 M Irgacure 2959, 3 M HEMA, 10 mL PBS solution; UV 4h).

D) Poly(acrylic acid) hydrogel (PAAc; 0.003 M MBAA, 0.004 M Irgacure 2959, 3 M AAc, 10 mL PBS solution; UV 4h).

E) Poly(vinyl alcohol) hydrogel (PVA; 2 g PVA, 8 mL PBS solution; F-T procedure three times).

1. **Measurement of gel-sol transition temperature**

The gel-sol transition temperature (T_t_) of the RPAD supramolecular hydrogel was determined using the following “falling steel ball method”. The RPAD 3.0% (w/v) was put into a glass bottle (inner diameter: 10 mm, height: 20 mm), sealed and was kept at room temperature for 6 h. A steel ball (diameter: 2 mm, weight: 100 mg) was put on the top of the hydrogel and the glass bottle was heated slowly in a thermostatic heating stage (heating rate: 1℃ min^-1^). T_t_ is defined as the temperature at which the ball reaches to the bottom of the glass bottle. An average of three such measurements was taken as T_t_. The temperature was determined in a reference vial filled with 2 mL of buffer solution.

1. **DSC analysis**

A differential scanning calorimeter (TA Instruments, DSC Q20) was used for the determination of the gel melting temperature of the RPAD gel. The gel sample was taken in a large-volume capsule (LVC) fitted with O-rings and was equilibrated at 0℃ for 15 min. It was then heated at a heating rate of 10℃ min^-1^ to 90℃ under a nitrogen atmosphere. It was then cooled at a rate of 10℃ min^-1^ to 0℃. The melting point and gelation temperature were determined from the peak position of the endotherm.

1. **Fourier transform infrared spectroscopy (FTIR)**

Fourier transform infrared spectroscopy (FTIR, Nicolet 6700, Thermo Fisher Scientific, USA) was used to record the chemical structure with the KBr pellet method. Absorbance spectra were collected over a range of 400-4000 cm^-1^.

1. **Static water contact angle (θ)**

All water contact angles were evaluated with a HARKE-SPCA (Harke, Beijing, China) at room temperature. A liquid droplet of 3 μL of DI water was dropped on the sample's surface, and the contact angle was calculated using the shadow technique. The values were based on three repetitions of the experiment.

**Formula S1**

In addition, the contact pressure between and contact ball can be calculated by Hertzian contact mechanism.[2, 3] As the pressure between contact ball and hydrogel surface is crucial to understanding the lubricating process, the contact pressure *p_0_*, contact radius *a*, and penetration depth *d* were calculated by Hertzian contact mechanism:

$p_{0}=\left( \frac{{6FE^{*}}^{2}}{\pi^{3}R^{2}} \right)^{1/3}$(1)

$a=\left( \frac{3FR}{4E^{*}} \right)^{1/3}$(2)

$d=\frac{\pi ap_{0}}{2E^{*}}$(3)

Here, *F* is the load, *R* is the radius of contact ball (6 mm), and *E** is the relevant modulus approximated by the low-frequency storage modulus G′. As shown in Table S1, the maximal contact pressure was 3.16 kPa with the load of 100 mN in our experiment.

**Formula S2**

The shear force (F_shear_) can be calculated as F_shear_ = σ·A. Here σ is shear stress that can be obtained by rheometer; A is the contact area. In our case the maximum contact area is 1.13 x 10^-4^ m^2^ , the shear force at this moment should be 4.18 mN.[1]

**Table S1.** Contact Pressure, Contact Radius, and Penetration Depth between PPRA-3 and Contact Ball.

| Load (mN) | 20 | 40 | 60 | 80 | 100 |
| --- | --- | --- | --- | --- | --- |
| Contact pressure (kPa) | 1.85 | 2.33 | 2.67 | 2.94 | 3.16 |
| Contact radius (mm) | 2.27 | 2.86 | 3.28 | 3.61 | 3.88 |
| Penetration depth (mm) | 1.03 | 1.64 | 2.15 | 2.60 | 3.00 |

**Table S2.** Comparison of self-prepared hydrogels with other switchable lubrication materials.

|  | Materials | External stimulus | COF of high friction state | COF of low friction state | Rate |
| --- | --- | --- | --- | --- | --- |
| Adv. Mater. 2023[4] | Hydrogel | Electrical | 0.12 | 0.06 | 2 |
| Macromol. Rapid. Comm. 2013[5] | Hydrogel | Thermal | 0.49 | 0.03 | 16.3 |
| Mater. Today. Chem. 2023[6] | Hydrogel | Humidity | 0.7 | 0.07 | 10 |
| Chinese. Chem. Lett. 2024[7] | Hydrogel | pH | 0.09 | 0.03 | 3 |
| ACS Appl. Polym.[8] Mater. 2021 | Hydrogel | Thermal | 0.75 | 0.33 | 2.3 |
| Eur. Polym. J. 2021[9] | Hydrogel | Salt | 0.5 | 0.03 | 16.7 |
| Tribol. Int. 2024[10] | Hydrogel | Thermal | 0.3 | 0.15 | 2 |
| J. Am. Chem. Soc. 2018[1] | Hydrogel | Shear | 0.06 | 0.02 | 3 |
| Matter. 2021[11] | Hydrogel | Light | 0.05 | 0.006 | 8.3 |
| This work | Hydrogel | Thermal or shear | 0.16 | 0.008 | 20 |

**Table S3.** The energy of AD, RP and RPAD.

| Model | Energy (kcal/mol, T = 293 K) | Energy (kcal/mol, T = 293 K, be sheared) | Energy (kcal/mol, T = 323 K) |
| --- | --- | --- | --- |
| AD | -293007.48 | -293007.48 | -293006.73 |
| RP | -1189722.24 | -1189722.24 | -1189721.18 |
| RPAD | -2965513.89 | -2965463.76 | -2965458.88 |

**Table S4.** Summary table of Abbreviations.

| Abbreviations | Full |
| --- | --- |
| PP | P(AAm-*co*-AAc)/PVA |
| PPRA | P(AAm-*co*-AAc)/PVA/RPAD |
| RPAD | Riboavin-5’-phosphate sodium salt-Adenine |
| PPRA-1  PPRA-3 | P(AAm-*co*-AAc)/PVA/RPAD (1 mg/mL)  P(AAm-*co*-AAc)/PVA/RPAD (3 mg/mL) |

**Supplementary figures:**

**
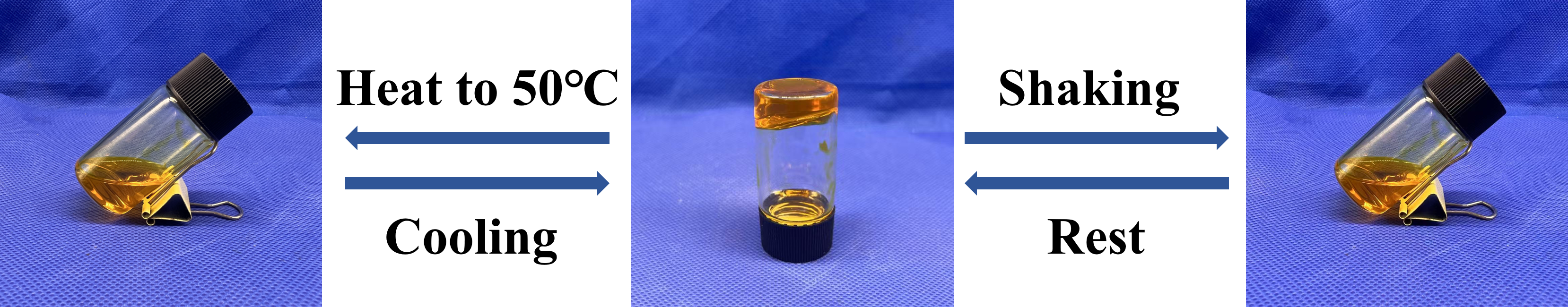
**

**Figure S1**. Photograph of the sol-gel transition of RPAD supramolecular hydrogel.


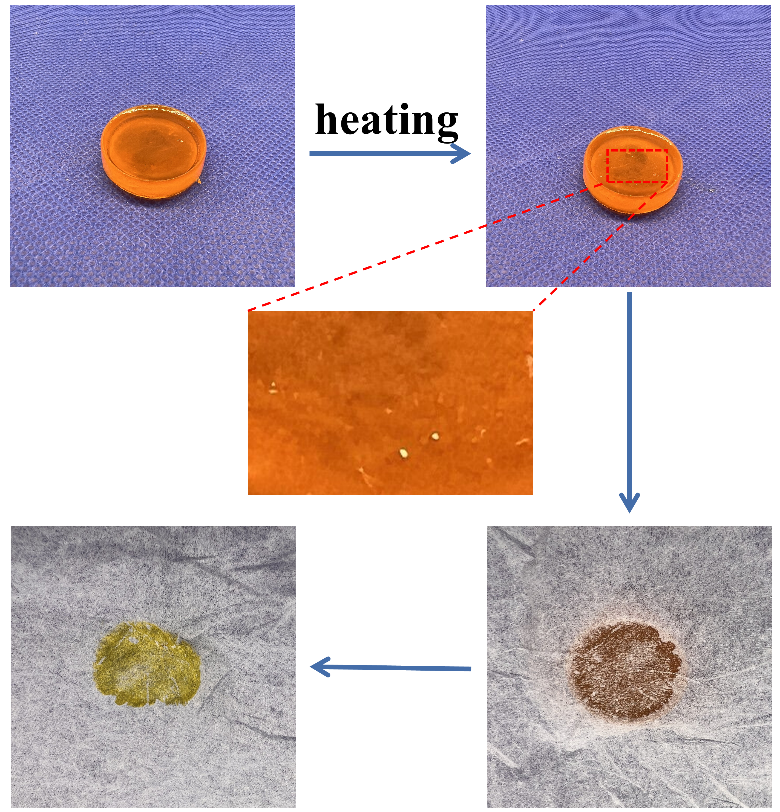


**Figure S2.** Photographs of the sol layer.


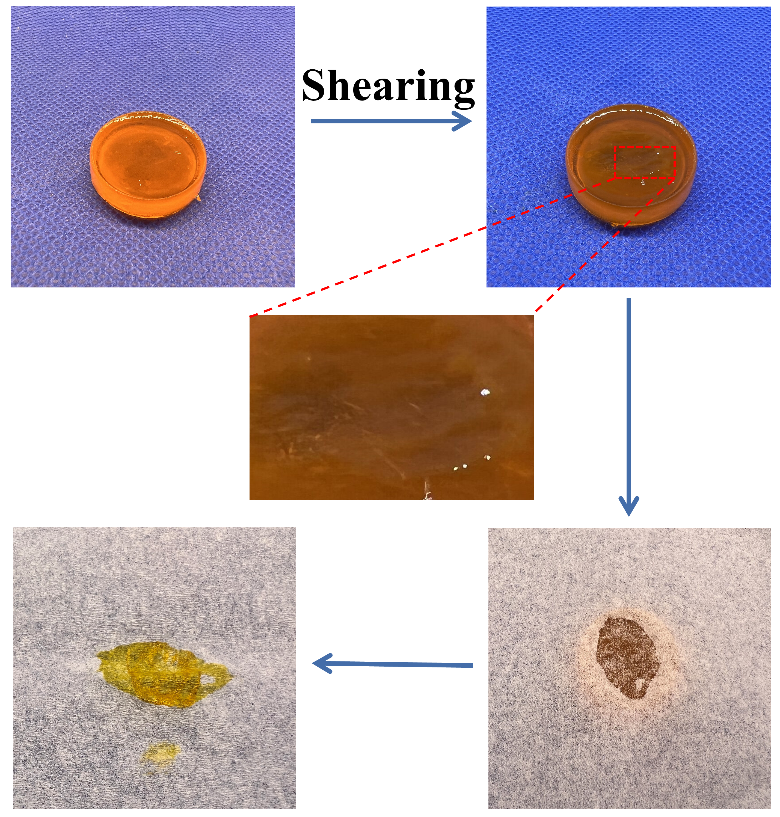


**Figure S3.** Photographs of the sol layer.


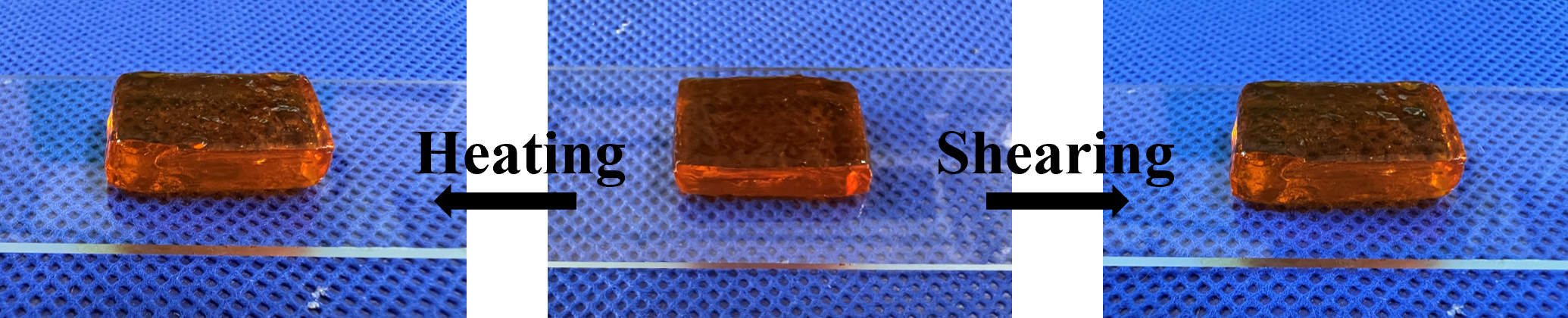


**Figure S4.** Photographs of the sol layer.


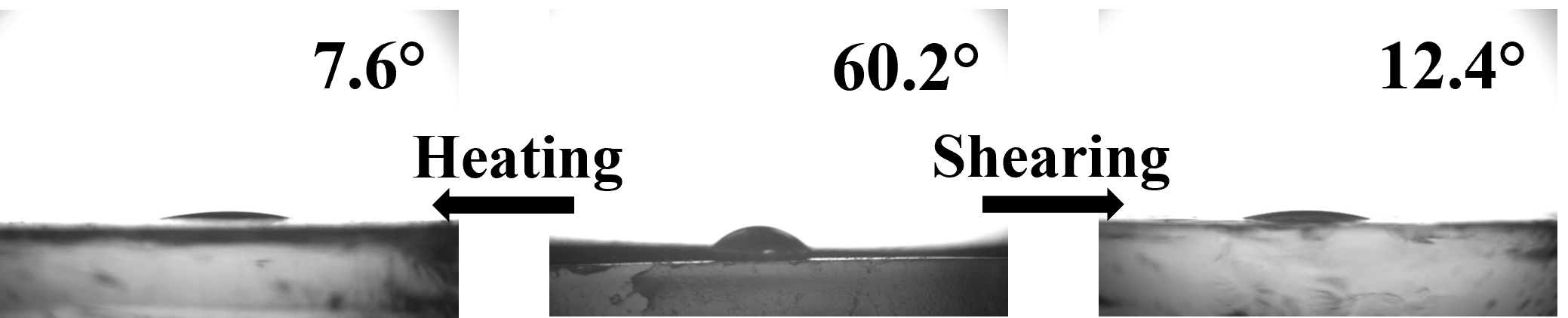


**Figure S5.** Corresponding contact angle images of Figure S3.

**
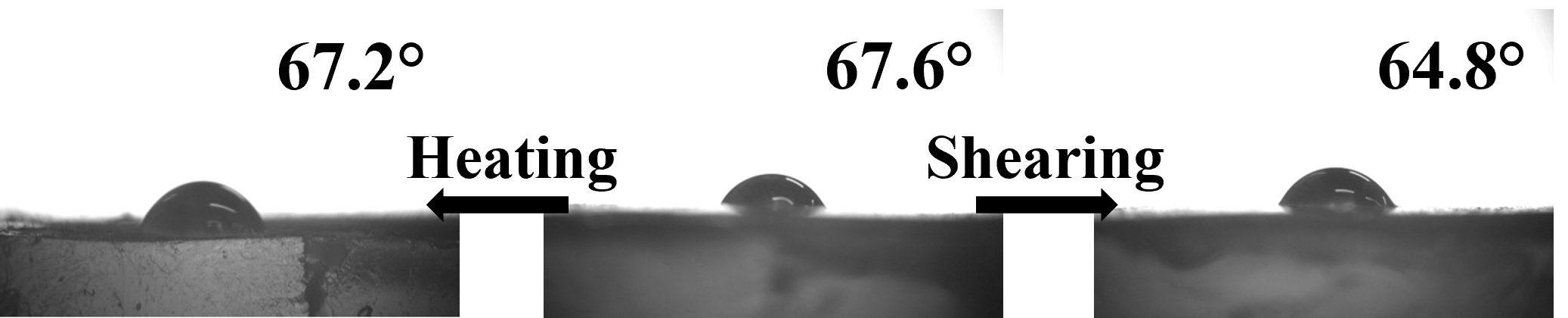
**

**Figure S6.** Contact angle images of P(AAm-*co*-AAc)/PVA hydrogel.

**
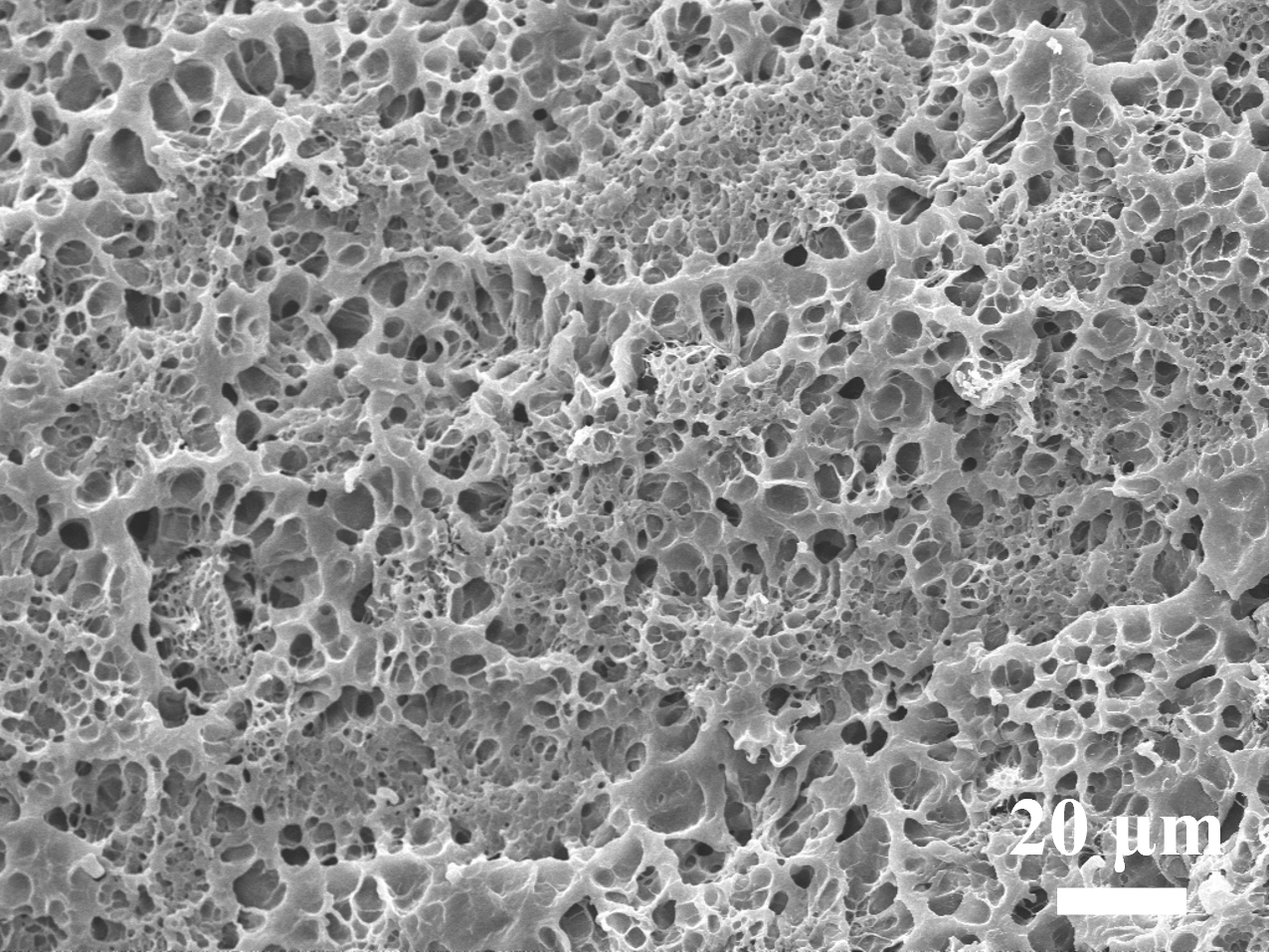
**

**Figure S7.** The SEM image of P(AAm-*co*-AAc)/PVA hydrogel.

**
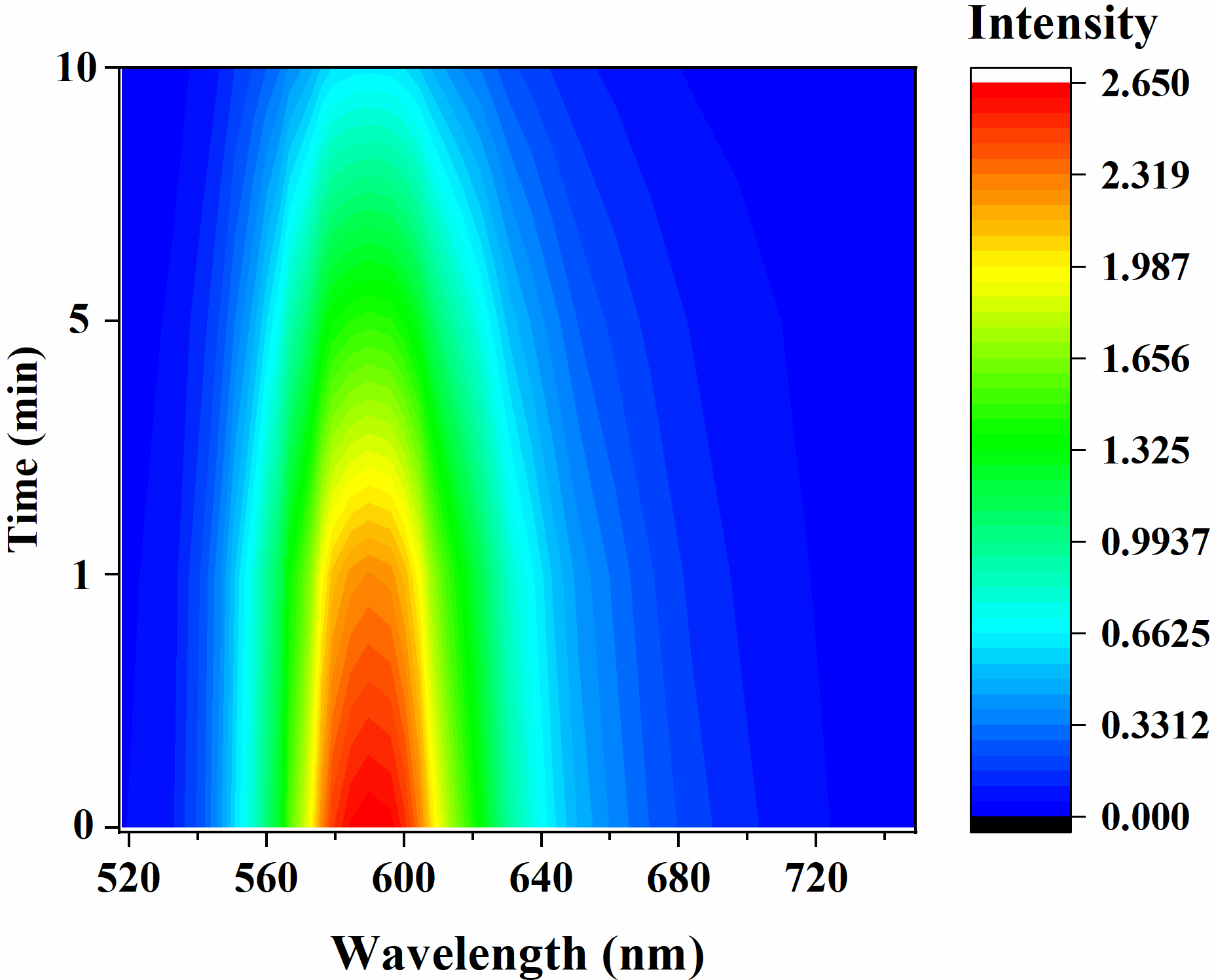
**

**Figure S8.** Fluorescence intensity of the sol layer extract on PPRA-3 as a function of the shearing time 0 to 10 min.


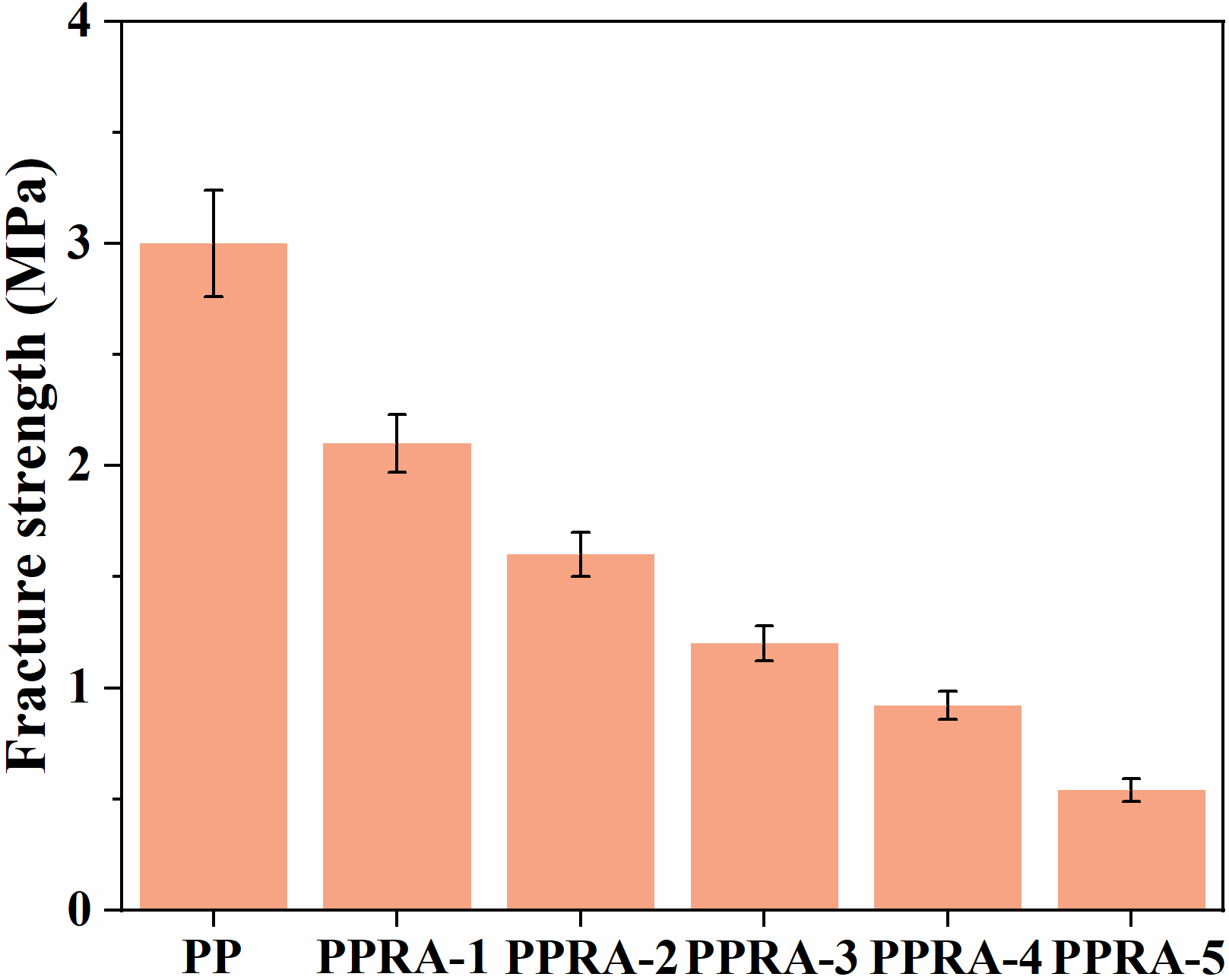


**Figure S9.** Fracture strength of the PP and PPRA hydrogels.


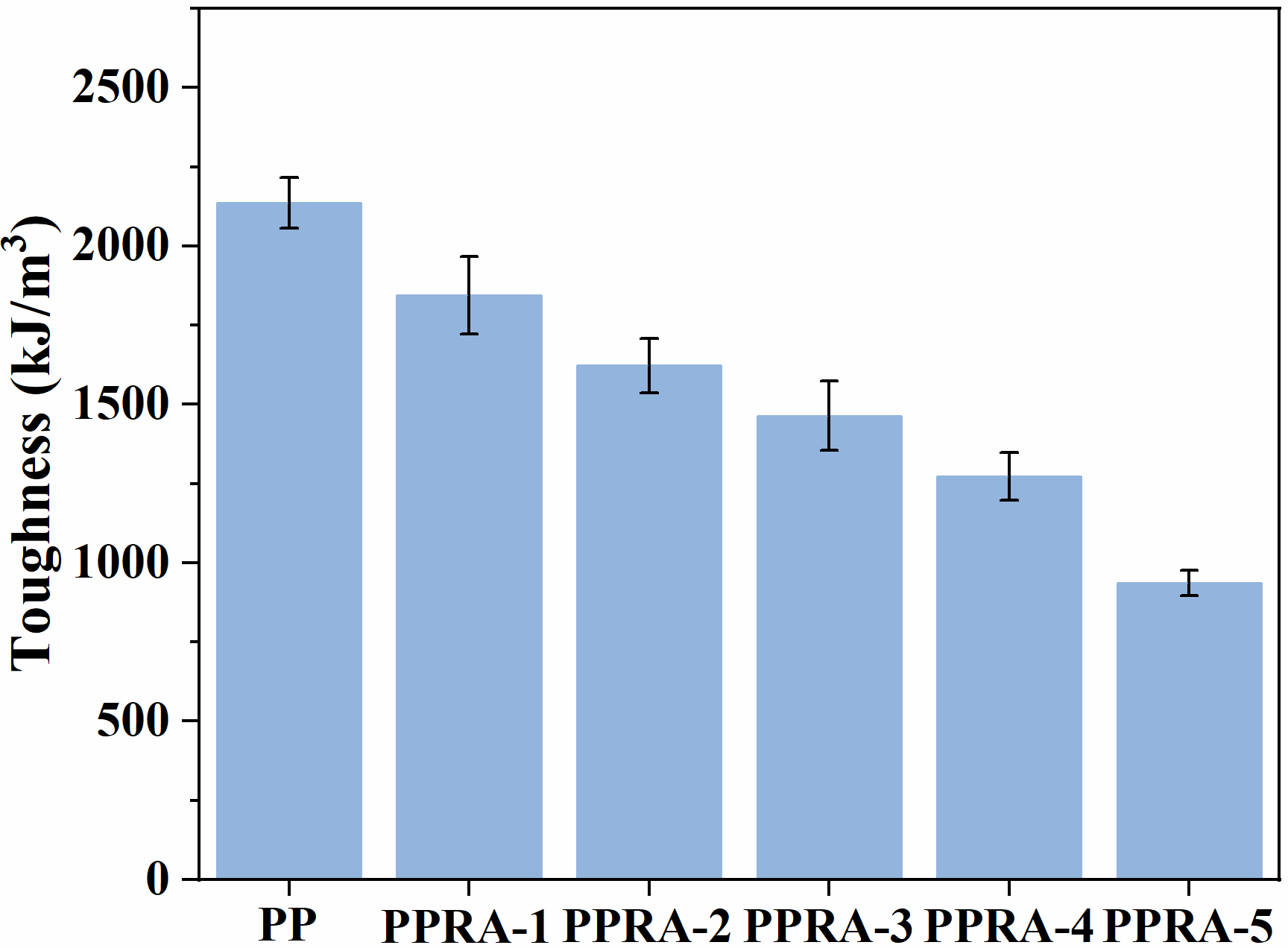


**Figure S10.** Toughness of the PP and PPRA hydrogels.


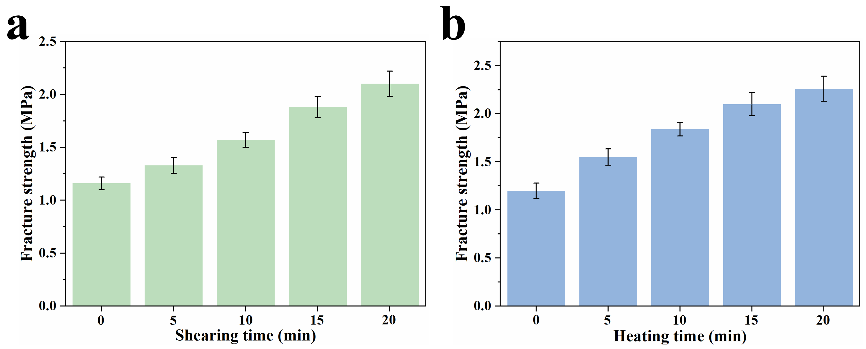


**Figure S11.** (a) Fracture strength of PPRA-3 under different shearing time, (b) fracture strength of PPRA-3 under different heating time.


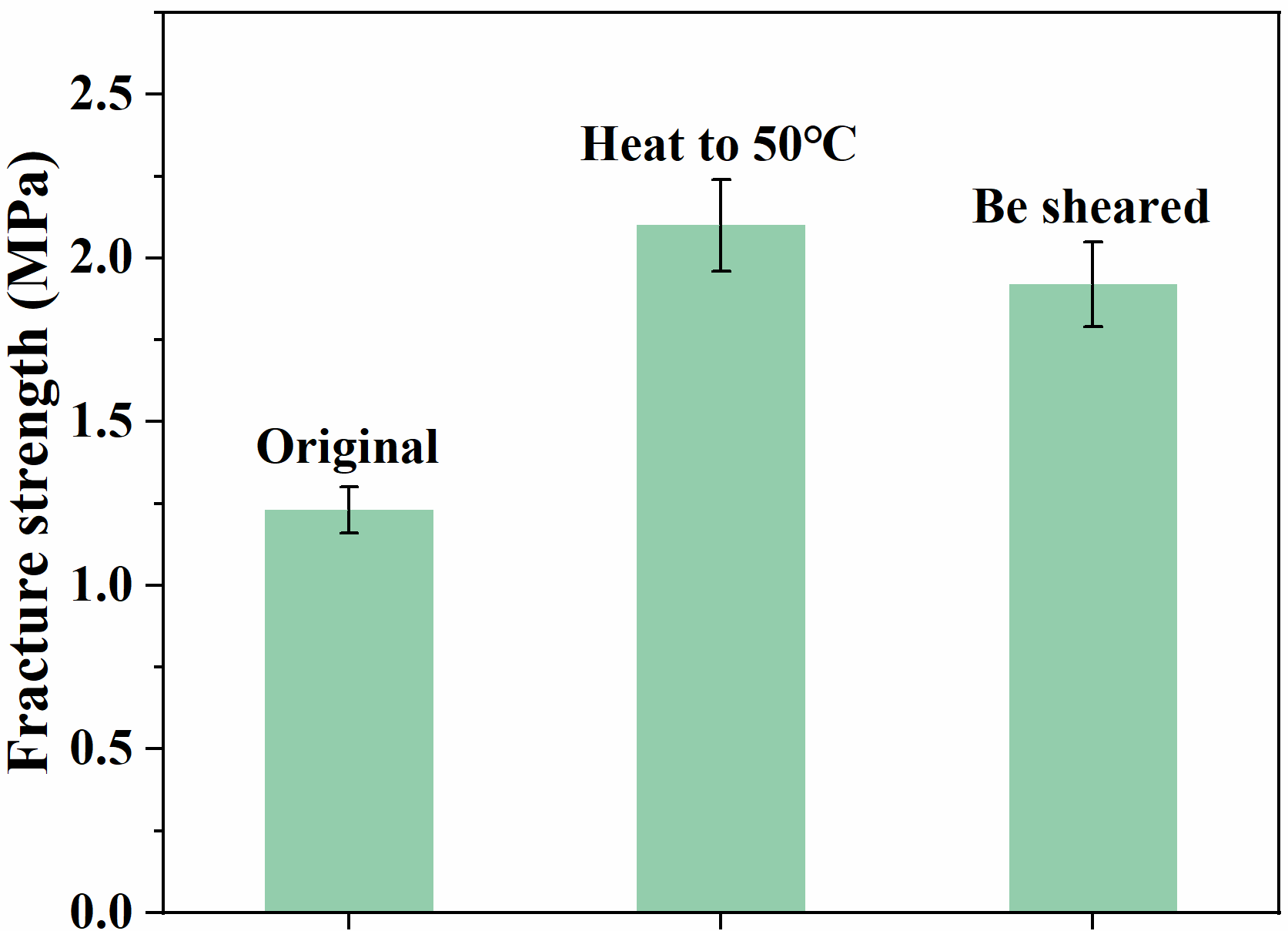


**Figure S12.** Fracture strength on original PPRA-3 hydrogel after being applied 20 shear cycles and after being heated for 10 min.


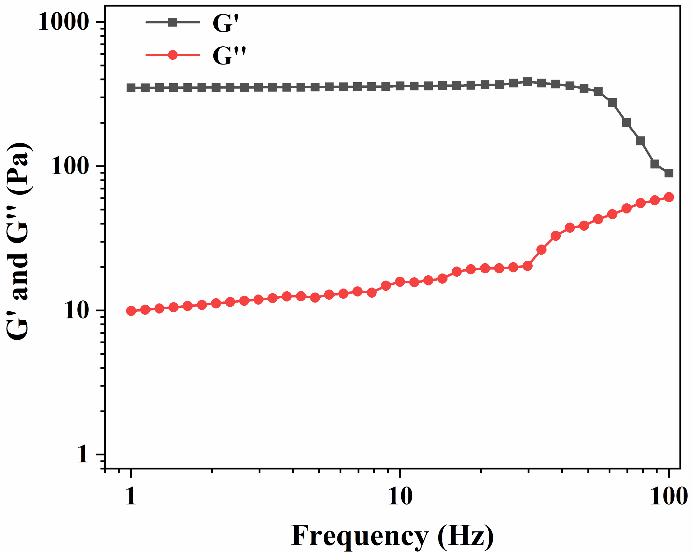


**Figure S13.** Frequency sweep data showing variation of storage modulus G′ and loss modulus G″ with frequency.


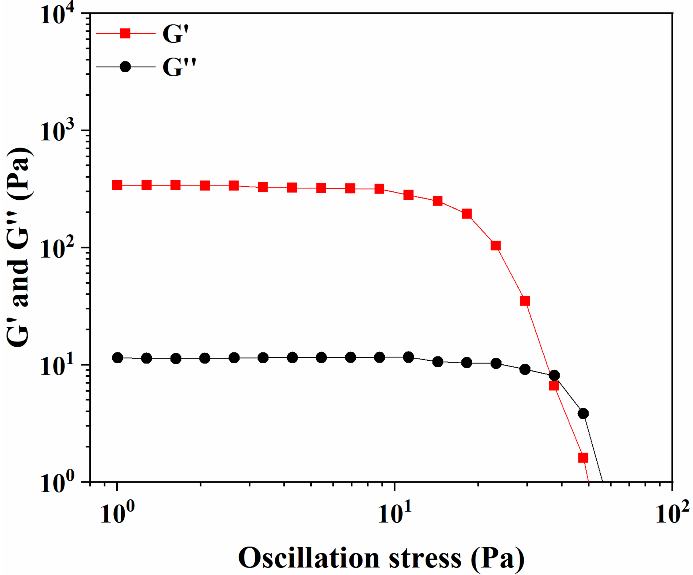

**Figure S14.** The oscillation stress dependency of the storage modulus G′ and loss modulus G″ of RPAD.

**
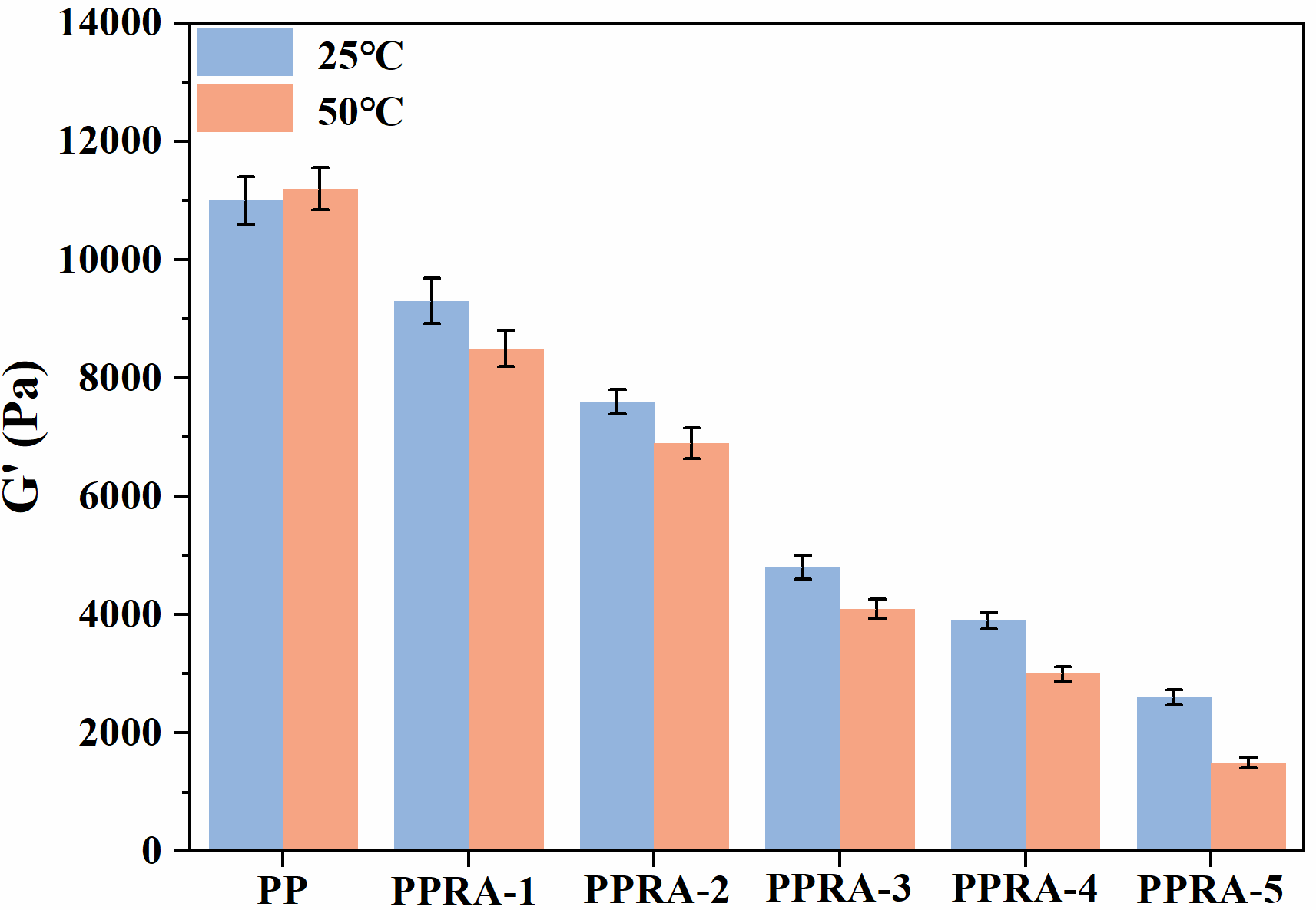
**

**Figure S15.** G′ of PVA/P(AAm-co-AAc) and PPRA hydrogels before and after being heated.

**
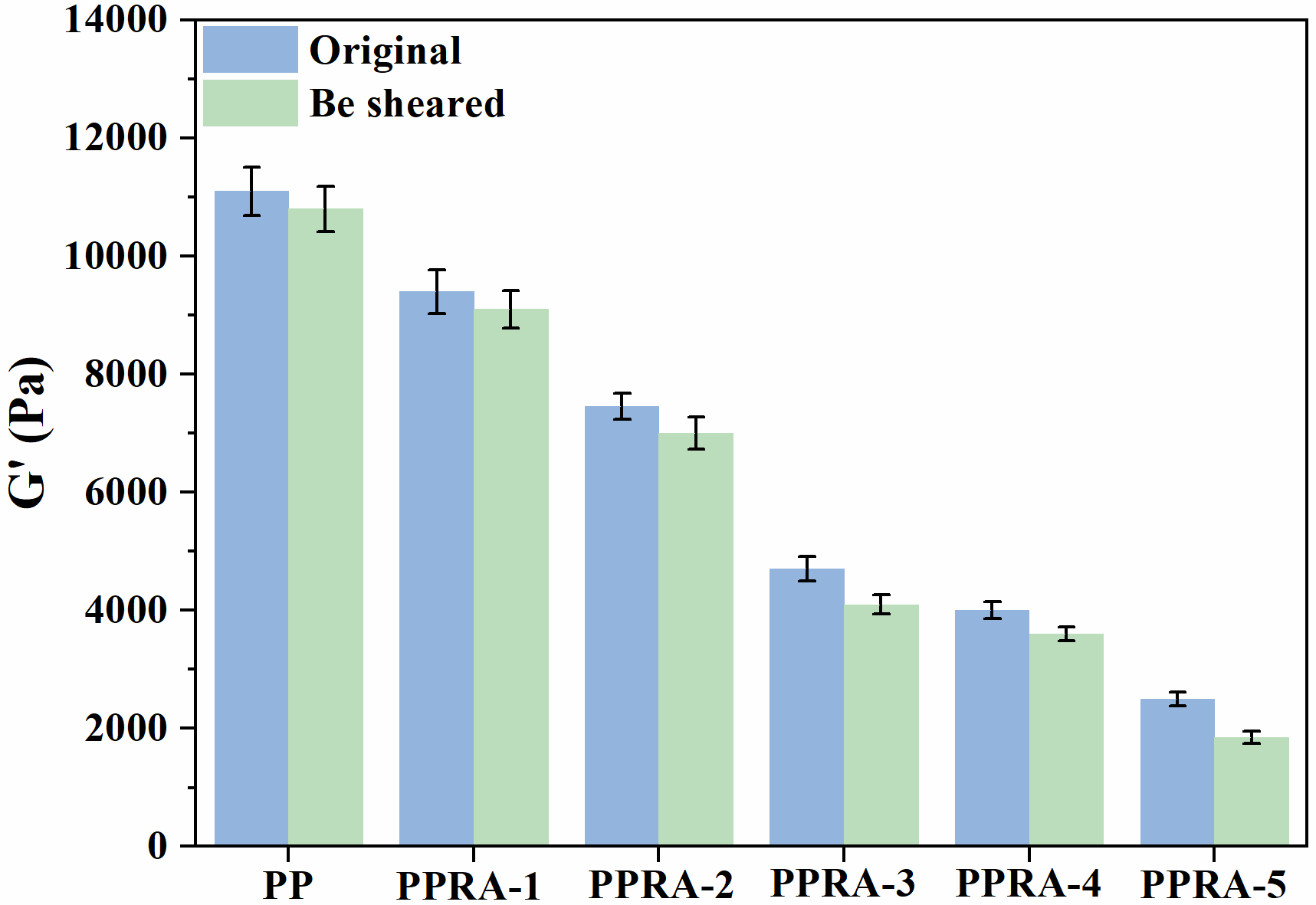
**

**Figure S16.** G′ of PVA/P(AAm-*co*-AAc) and PPRA hydrogels before and after being sheared.


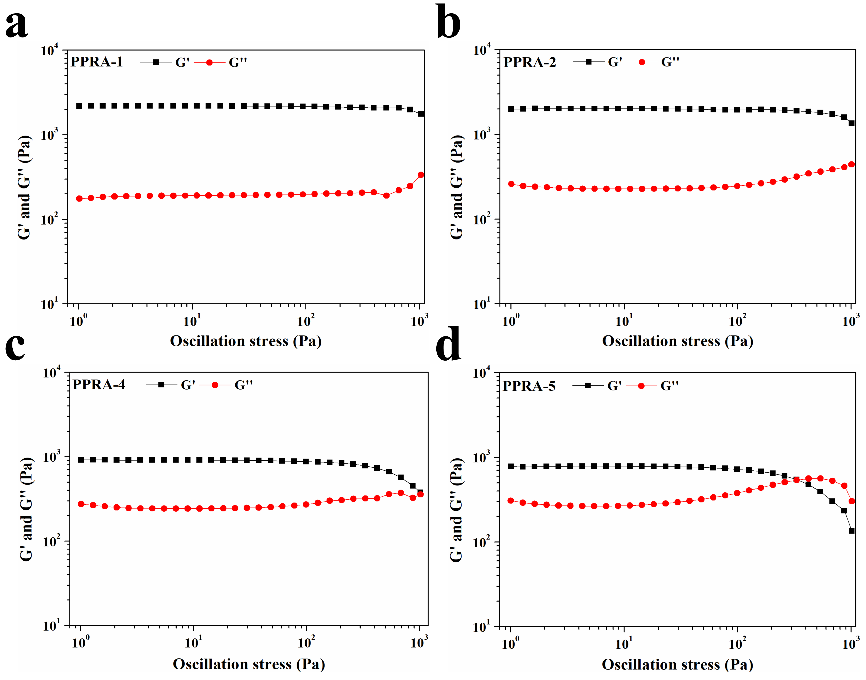


**Figure S17.** The oscillation stress dependency of the storage modulus G′ and loss modulus G″ of PPRA hydrogels.

**
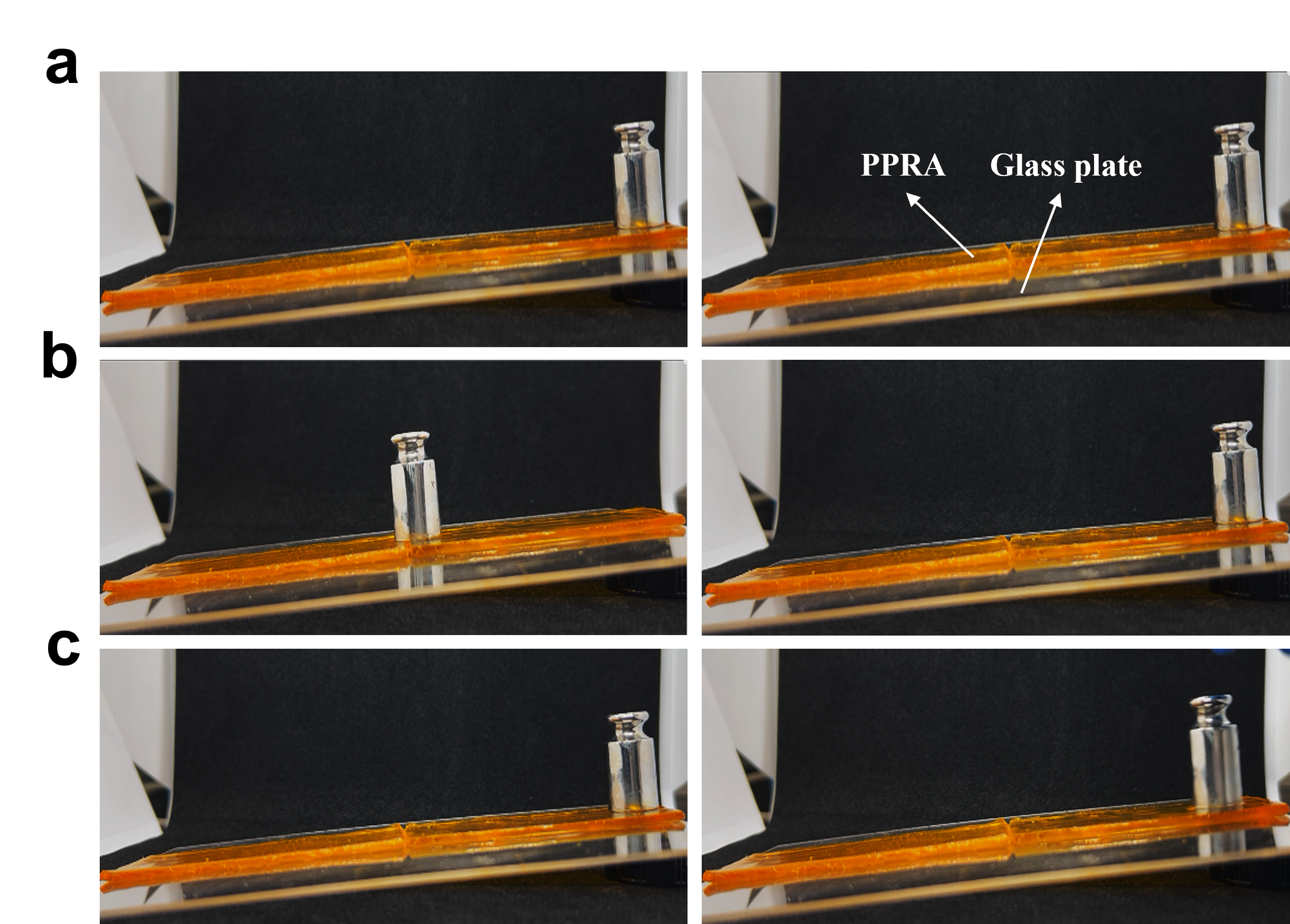
**

**Figure S18.** Shear-responsive lubricating demonstration of PPRA hydrogel.


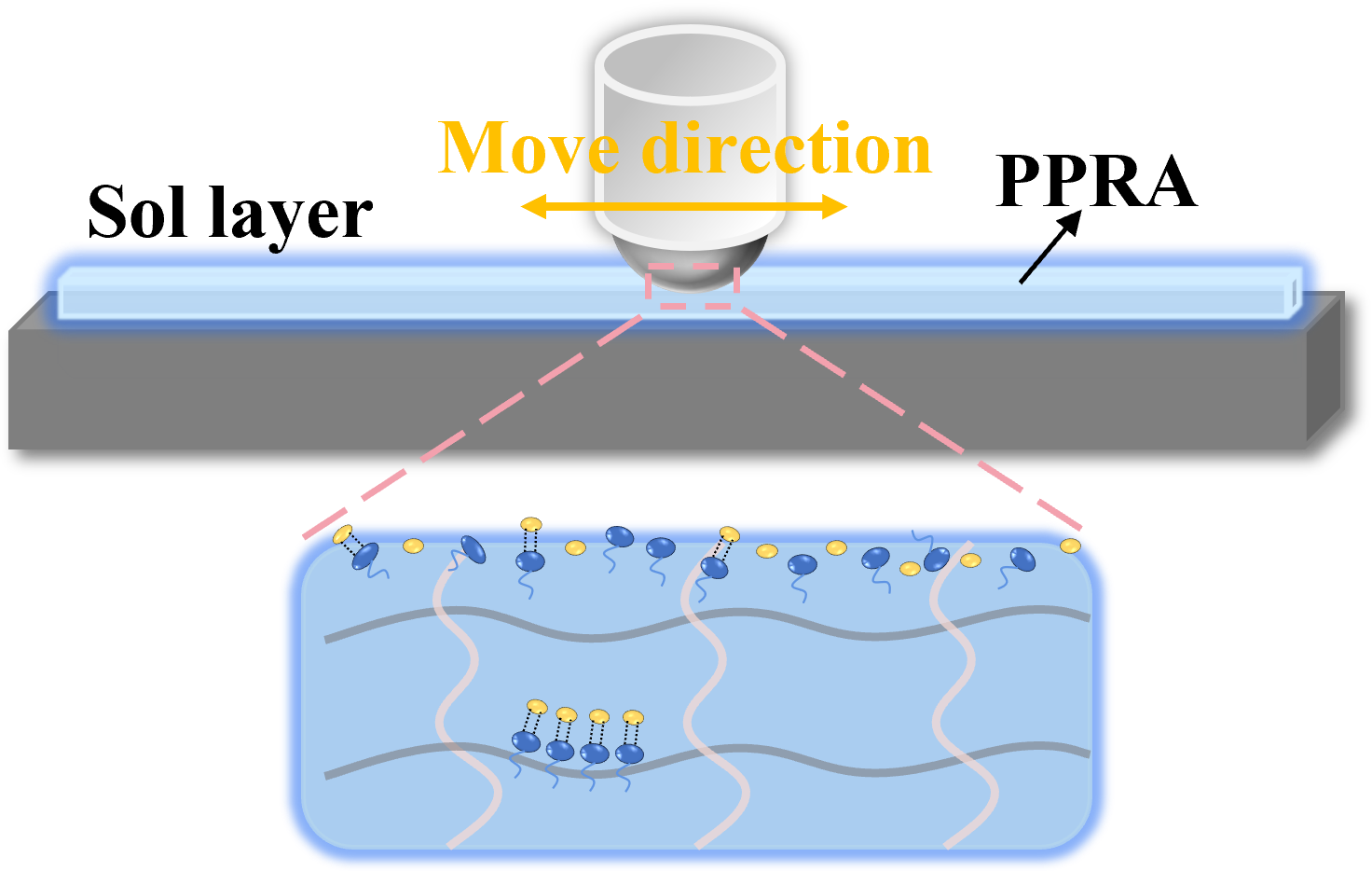


**Figure S19.** Schematic diagram of shear-responsive test.


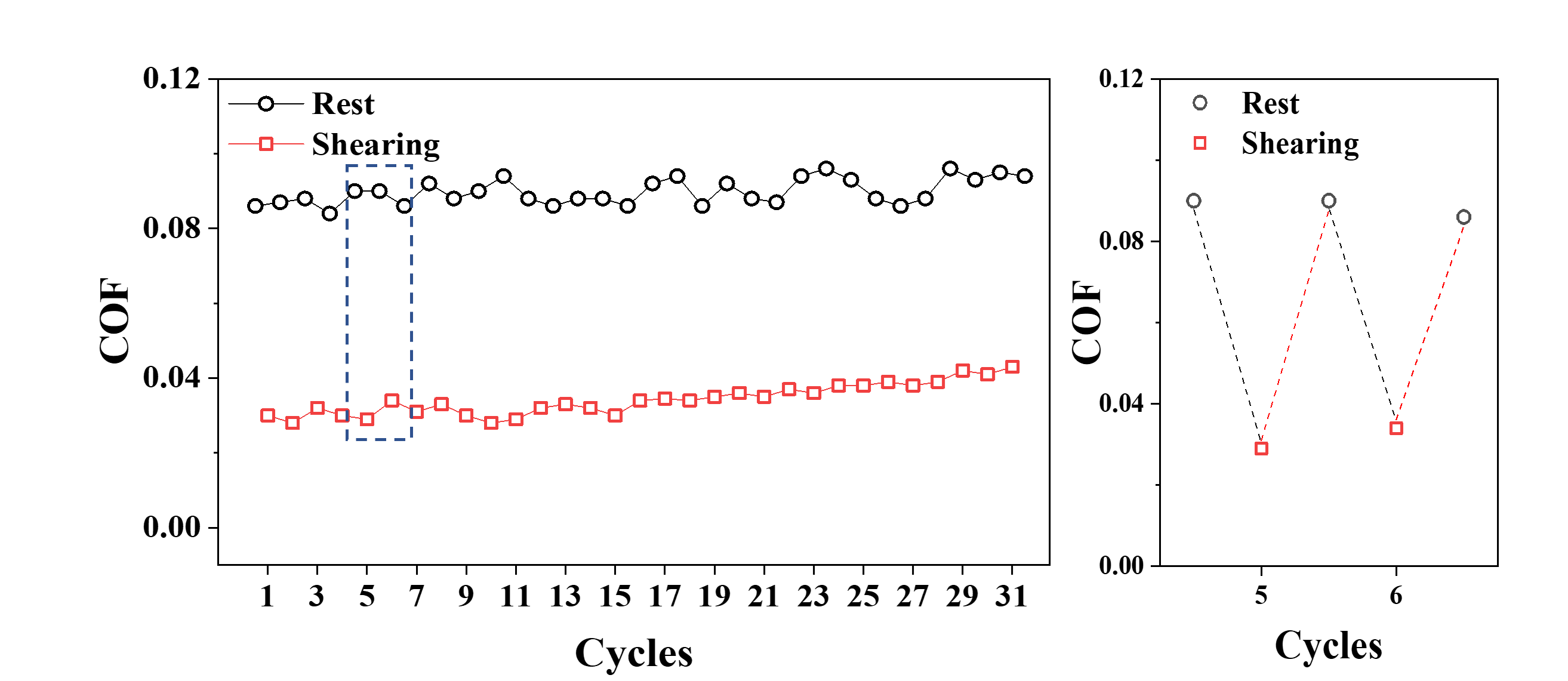


**Figure S20.** Reversibility of shear-responsive lubrication on PPRA-3.

**
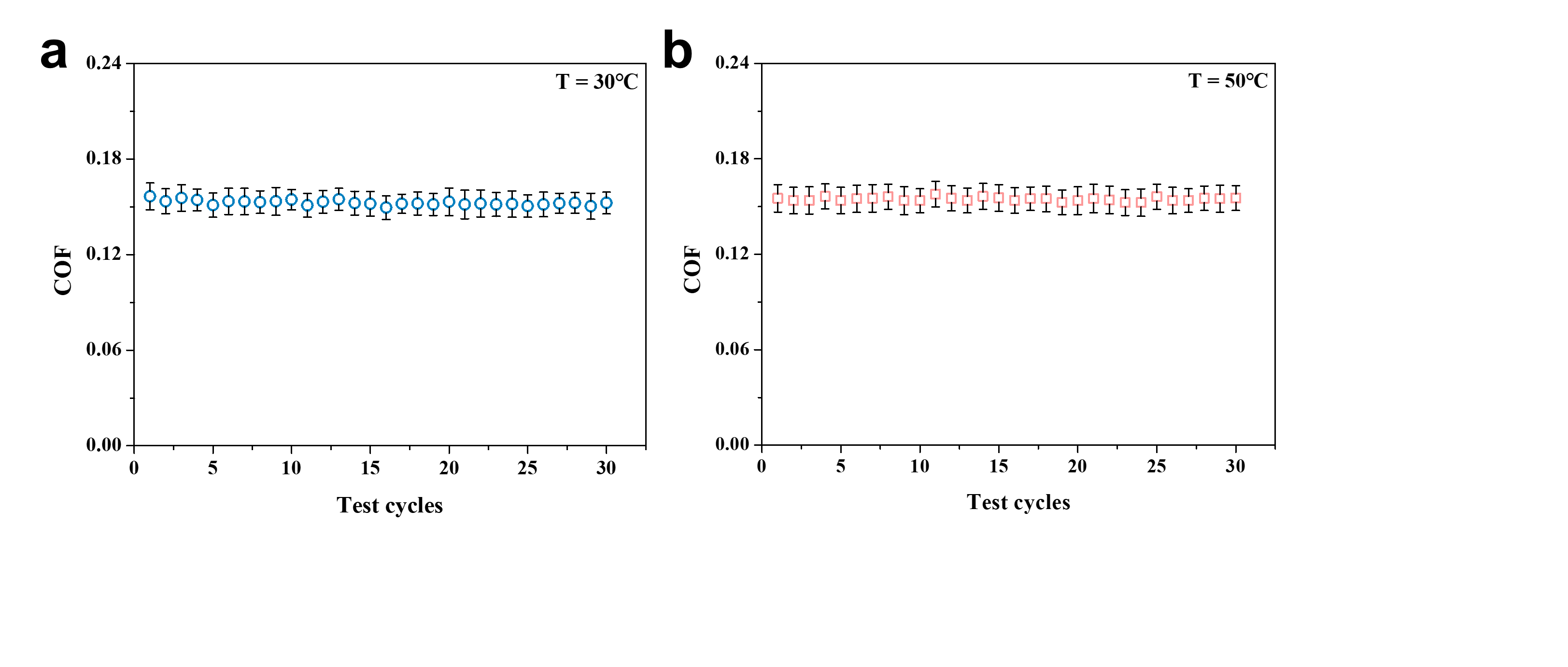
**

**Figure S21.** The COFs on the P(AAm-*co*-AAc)/PVA hydrogels following 30 shear cycles.


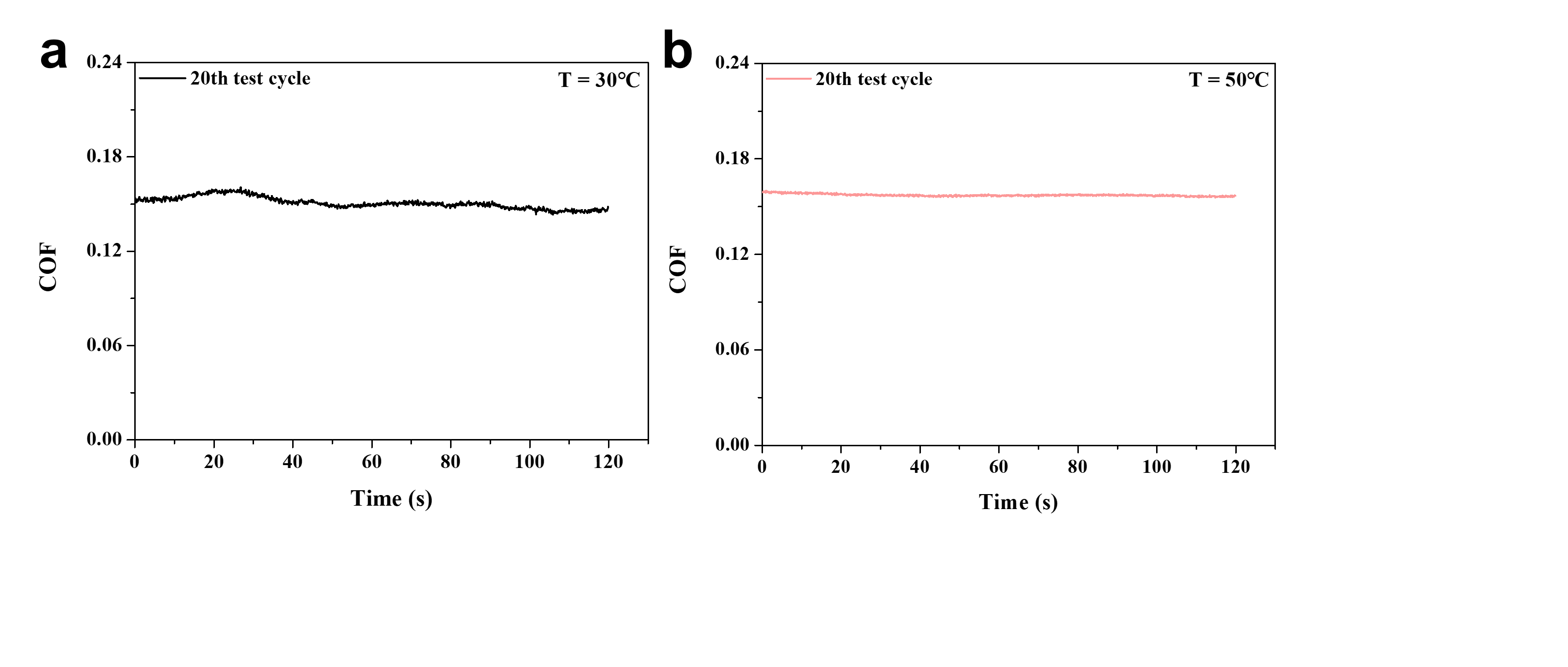


**Figure S22.** The COFs on the P(AAm-*co*-AAc)/PVA hydrogels surface at 20th test cycle.

**
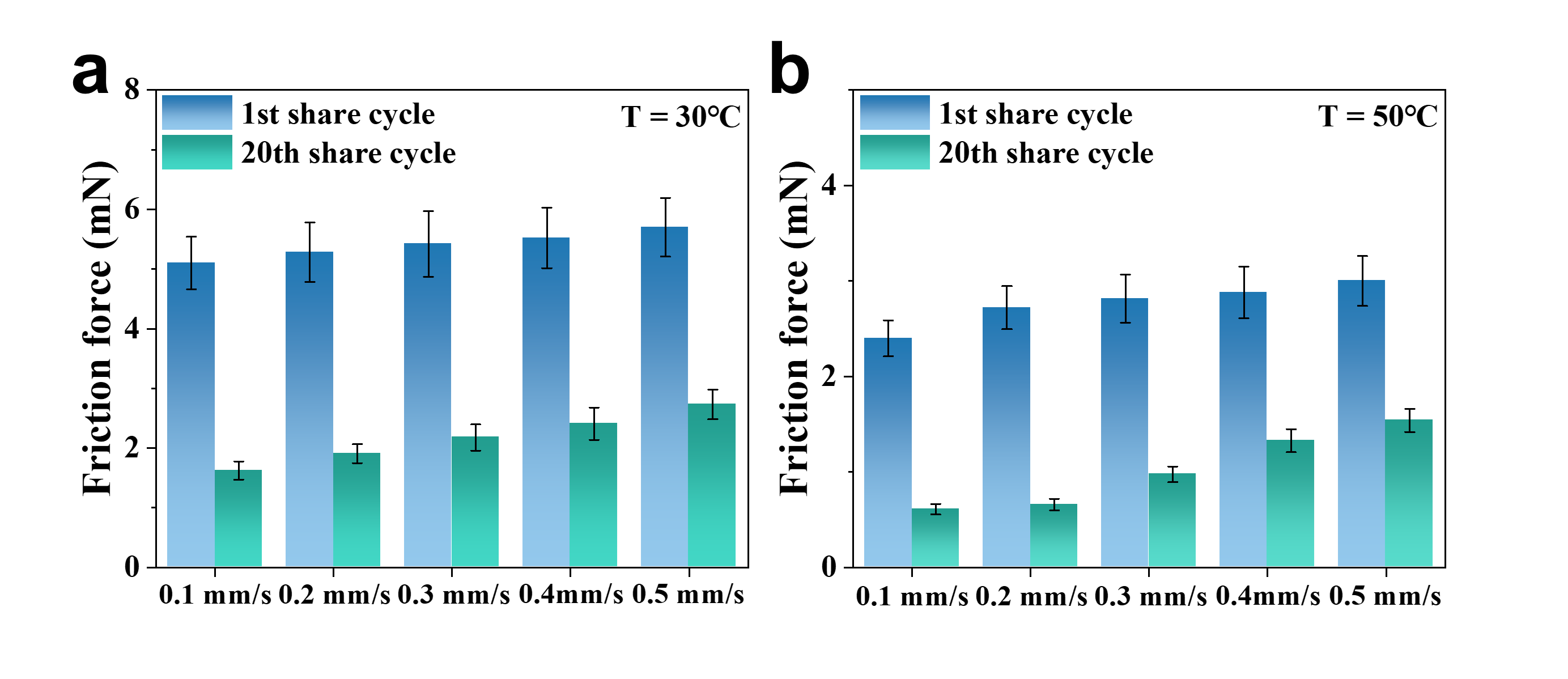
**

**Figure S23.** (a) (b) Keeping the load at 60 mN, relationship between the friction force on PPRA-3 under different sliding velocities of 0.1- 0.5 mm/s.


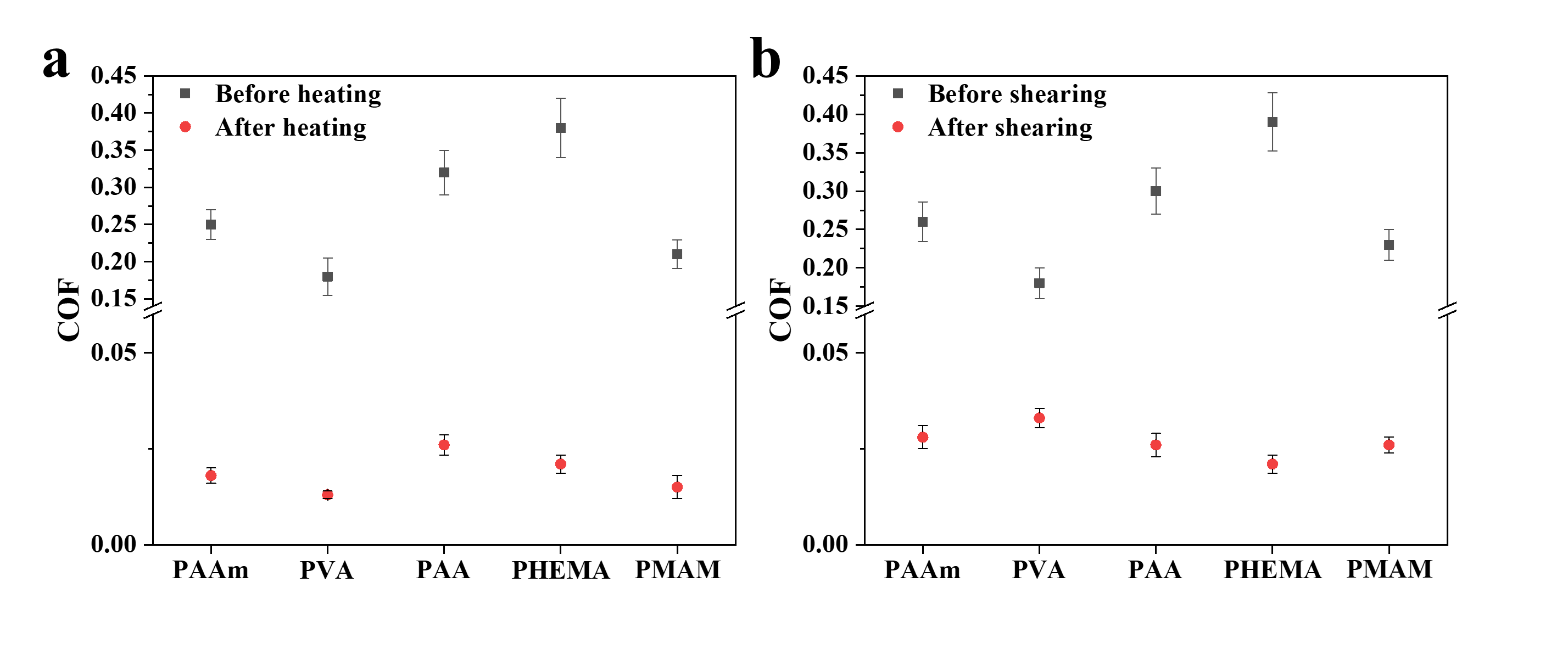


**Figure S24.** COFs of different thermal/shear-responsive hydrogels.


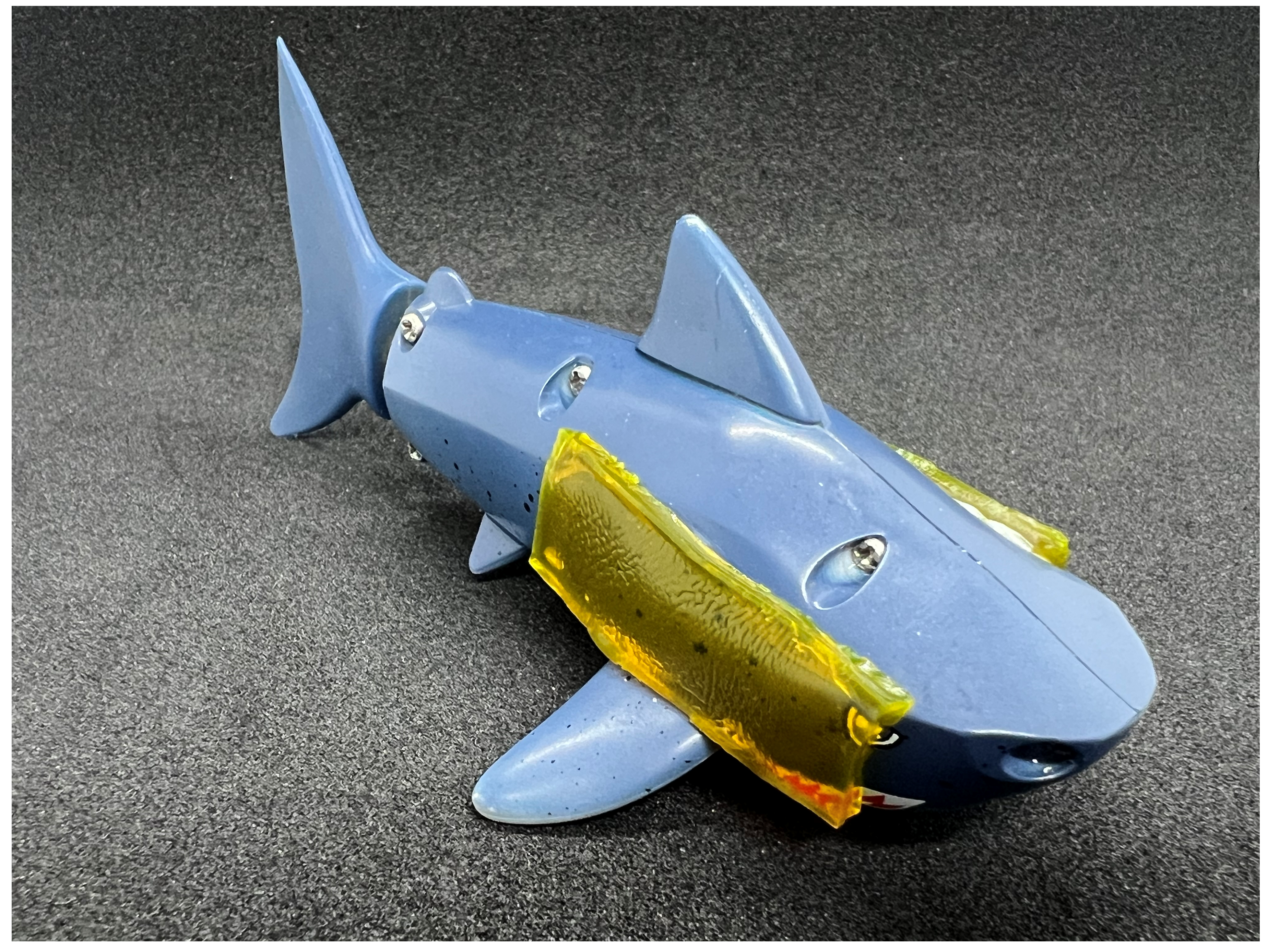


**Figure S25.** Photograph of the electric fish with PPRA hydrogels.

References

[1] X. Zhang, J. Wang, H. Jin, S. Wang, W. Song, Bioinspired Supramolecular Lubricating Hydrogel Induced by Shear Force, Journal of the American Chemical Society, 140 (2018) 3186-3189.

[2] A.C. Fischer-Cripps, The Hertzian contact surface, Journal of Materials Science, 34 (1999) 129-137.

[3] J. Gong, Y. Iwasaki, Y. Osada, K. Kurihara, Y. Hamai, Friction of Gels. 3. Friction on Solid Surfaces, The Journal of Physical Chemistry B, 103 (1999) 6001-6006.

[4] J. Kang, X. Zhang, X. Yang, X. Yang, S. Wang, W. Song, Mucosa-Inspired Electro-Responsive Lubricating Supramolecular-Covalent Hydrogel, Advanced Materials, 35 (2023) 2307705.

[5] Y. Wu, M. Cai, X. Pei, Y. Liang, F. Zhou, Switching Friction with Thermal- Responsive Gels, Macromolecular Rapid Communications, 34 (2013) 1785-1790.

[6] Q. Wang, P. Cao, Y. Gao, J. Yang, Z. Lu, T. Wang, Y. Zhang, Dynamic hydrogen bonding induced hygroscopicity of ionogel enabling lubricating regulation, Materials Today Chemistry, 34 (2023) 101762.

[7] J. Kang, X. Yang, X. Yang, J. Sun, Y. Liu, S. Wang, W. Song, Carbon dots-enhanced pH-responsive lubricating hydrogel based on reversible dynamic covalent bondings, Chinese Chemical Letters, 35 (2024) 109297.

[8] Z. Chen, Y. Feng, N. Zhao, J. Shi, G. Liu, W. Liu, Near-Infrared Photothermal Microgel for Interfacial Friction Control, ACS Applied Polymer Materials, 3 (2021) 4055-4061.

[9] S. Xiao, X. He, Z. Zhao, G. Huang, Z. Yan, Z. He, Z. Zhao, F. Chen, J. Yang, Strong anti-polyelectrolyte zwitterionic hydrogels with superior self-recovery, tunable surface friction, conductivity, and antifreezing properties, European Polymer Journal, 148 (2021) 110350.

[10] Y. Huang, Z. Li, K. Hou, S. Liu, Q. Gao, X. Miao, J. Wang, S. Yang, Photothermally responsive and durable polydopamine-modified MXene-PNIPAM hydrogels for smart friction regulation, Tribology International, 193 (2024) 109435.

[11] J. Wang, X. Zhang, S. Zhang, J. Kang, Z. Guo, B. Feng, H. Zhao, Z. Luo, J. Yu, W. Song, S. Wang, Semi-convertible Hydrogel Enabled Photoresponsive Lubrication, Matter, 4 (2021) 675-687.
